# ^18^F-FDG PET as an imaging biomarker for the response to FGFR-targeted therapy of cancer cells via FGFR-initiated mTOR/HK2 axis

**DOI:** 10.7150/thno.74848

**Published:** 2022-08-29

**Authors:** Yuchen Jiang, Qinghe Zeng, Qinghui Jiang, Xia Peng, Jing Gao, Haiyan Wan, Luting Wang, Yinglei Gao, Xiaoyu Zhou, Dongze Lin, Hanyi Feng, Sheng Liang, Hu Zhou, Jian Ding, Jing Ai, Ruimin Huang

**Affiliations:** 1 School of Pharmacy, Nanchang University, Nanchang 330006, China.; 2 Division of Antitumor Pharmacology, State Key Laboratory of Drug Research, Shanghai Institute of Materia Medica, Chinese Academy of Sciences, Shanghai 201203, China.; 3 Molecular Imaging Center, Shanghai Institute of Materia Medica, Chinese Academy of Sciences, Shanghai 201203, China.; 4 University of Chinese Academy of Sciences, Beijing 100049, China.; 5 Analytical Research Center for Organic and Biological Molecules, Shanghai Institute of Materia Medica, Chinese Academy of Sciences, Shanghai 201203, China.; 6 CAS Key Laboratory of Receptor Research, Shanghai Institute of Materia Medica, Chinese Academy of Sciences, Shanghai 201203, China.; 7 School of Chinese Materia Medica, Nanjing University of Chinese Medicine, Nanjing 210023, China.; 8 Department of Nuclear Medicine, Xinhua Hospital, School of Medicine, Shanghai Jiao Tong University, Shanghai 200092, China.

**Keywords:** ^18^F-FDG, FGFR, Therapeutic Response, PET/CT, mTOR/HK2

## Abstract

**Rationale:** The overall clinical response to FGFR inhibitor (FGFRi) is far from satisfactory in cancer patients stratified by FGFR aberration, the current biomarker in clinical practice. A novel biomarker to evaluate the therapeutic response to FGFRi in a non-invasive and dynamic manner is thus greatly desired.

**Methods:** Six FGFR-aberrant cancer cell lines were used, including four FGFRi-sensitive ones (NCI-H1581, NCI-H716, RT112 and Hep3B) and two FGFRi-resistant ones (primary for NCI-H2444 and acquired for NCI-H1581/AR). Cell viability and tumor xenograft growth analyses were performed to evaluate FGFRi sensitivities, accompanied by corresponding ^18^F-fluorodeoxyglucose (^18^F-FDG) uptake assay. mTOR/PLCγ/MEK-ERK signaling blockade by specific inhibitors or siRNAs was applied to determine the regulation mechanism.

**Results:** FGFR inhibition decreased the *in vitro* accumulation of ^18^F-FDG only in four FGFRi-sensitive cell lines, but in neither of FGFRi-resistant ones. We then demonstrated that FGFRi-induced transcriptional downregulation of hexokinase 2 (HK2), a key factor of glucose metabolism and FDG trapping, via mTOR pathway leading to this decrease. Moreover, ^18^F-FDG PET imaging successfully differentiated the FGFRi-sensitive tumor xenografts from primary or acquired resistant ones by the tumor ^18^F-FDG accumulation change upon FGFRi treatment. Of note, both ^18^F-FDG tumor accumulation and HK2 expression could respond the administration/withdrawal of FGFRi in NCI-H1581 xenografts correspondingly.

**Conclusion:** The novel association between the molecular mechanism (FGFR/mTOR/HK2 axis) and radiological phenotype (^18^F-FDG PET uptake) of FGFR-targeted therapy was demonstrated in multiple preclinical models. The adoption of ^18^F-FDG PET biomarker-based imaging strategy to assess response/resistance to FGFR inhibition may benefit treatment selection for cancer patients.

## Introduction

Tyrosine kinase receptor fibroblast growth factor receptors (FGFRs) consist of 4 members including FGFR1, 2, 3, and 4, which play critical and diverse roles in early embryonic development and maintaining body metabolic balance. FGFR aberrant activation via gene fusion, activating mutation and amplification as well as ligand stimulation can promote tumor initiation and development in a variety of cancers 1-3. For example, FGFR1 amplification was found in approximately 6% of lung cancer cases, mainly in squamous non-small cell lung carcinoma subtype without effective treatments 1,4. FGFR2 was amplified in less than 10% of gastric cancer cases, associated with bad prognosis 1,2. FGFR2 fusion occurred in 45% of intrahepatic cholangiocarcinoma cases 5. Notably, fusion and activating mutations of FGFR3 frequently occurred in urothelial bladder carcinomas and predominantly in non-muscle invasive urothelial cell carcinoma type (occurring in 75% of cases) 2,6,7. FGF19 amplification-induced FGFR4 activation was observed in hepatocellular carcinomas and might represent FGFR4-dependent cancer subtype 1,2,8-10. FGFRs thus become attractive targets for anti-cancer drug development. The pan-FGFR inhibitor (FGFRi) Erdafitinib, active against FGFR1-4, is the first FGFRi approved by FDA to treat patients with locally advanced or metastatic urothelial carcinoma with susceptible FGFR3 or FGFR2 genetic alterations, which has progressed during or following platinum-containing chemotherapy in 2019 11,12. Immediately afterward, FDA granted the approvals of FGFR1-3 inhibitors (Pemigatinib and Infigratinib) for patients with previously treated, unresectable locally advanced or metastatic cholangiocarcinoma with FGFR2 fusion or other rearrangements in 2020 13 and in 2021 14, respectively. Besides these three approved inhibitors, numerous FGFRi inhibitors, such as Rogaratininb, AZD4547, and Futibatinib, are still in phase I-III clinical trials in various malignancies 15,16.

Despite a promising prospect of FGFRi in certain cancer patients, its clinical efficacy is far from satisfactory with an overall response rate of 20-40% in the approved indication 17-19. Even much lower response rate was observed in other cancer types 1,15,20. Primary and acquired resistances to FGFR therapy due to the secondary mutations and the feedback activation of alternate pathways may decrease their clinical benefits 1,15. It is indicated that the patients stratifying strategy based on FGFR aberration alone is very limited and cannot guarantee the patients' response to FGFRi. Exploring the therapeutic response biomarkers of FGFRi is therefore an urgent need. We have identified c-Myc, a fundamental downstream effector of FGFR signaling, could determine the therapeutic response to FGFRi in FGFR-addicted cancers 21. Usually, c-Myc expression levels are examined in the tumor samples via biopsy or surgery, as well as in the circulating tumor cells (CTCs). However, sample availability, sensitivity on CTCs-based assays and tumor heterogenicity are unable to guarantee the accurate assessment of c-Myc. A novel biomarker to evaluate the therapeutic response to FGFRi in patients in a non-invasive, real-time and quantitative manner is greatly desired.

The identification of several FGFs, such as FGF1, FGF15/19, FGF21 and FGF23, with high relevance to metabolic regulation 22-25 attracted our attention. In fact, deregulating cellular metabolism, the hallmark of cancer, is required by the tumor cells to meet energy and structural requirements for rapid proliferation 26,27. Notably, these metabolic changes are indispensable for certain cancers, making such tumors with metabolic vulnerability 26; therefore, they can be exploited as therapeutic intervention or monitoring therapeutic response. We noticed that aberrant FGFR1 could enhance the Warburg Effect to drive prostate cancer progression by reprogramming LDH isoform expression and activity 28. Meanwhile, the biologic basis for ^18^F-fluorodeoxyglucose (^18^F-FDG) positron emission tomography (PET) is the Warburg Effect 29. ^18^F-FDG is a radiolabeled analogue of glucose whereby the 2' hydroxyl group is substituted with ^18^F. FDG passes the cellular membrane mediated by the glucose transporters (GLUTs) and is phosphorylated by hexokinases (HKs) to FDG-6-phosphate, which cannot be further metabolized to participate the tricarboxylic acid cycle 30. Dephosphorylation of FDG-6-phosphate back to FDG by glucose-6-phosphatase is the only way to exit the cells. The enhanced levels of GLUTs and HKs, along with the reduced level of glucose-6-phosphatase in tumors lead to FDG trapping in cancer cells 31. ^18^F-FDG PET imaging has thus been most widely applied in clinical practice and become the gold standard for oncology 32.

Considering the huge clinical translation potential, we investigated in this study whether ^18^F-FDG PET could be used as a biomarker candidate for the therapeutic response to FGFRi in oncology. In different preclinical tumor models *in vitro* and *in vivo*, we found FGFR-targeted therapy decreased the accumulation of ^18^F-FDG only in FGFRi-sensitive tumors, but not in FGFRi-resistant ones. We then demonstrated that downregulation of hexokinase 2 (HK2) via mTOR pathway by FGFR inhibition leading to the decrease of ^18^F-FDG uptake in FGFRi-sensitive cells. A novel application of the well-established ^18^F-FDG PET imaging to functional assessment of the treatment response to FGFR-targeted therapy in cancer patients as well as the underlying molecular mechanism are suggested.

## Methods

### Cell culture and reagents

NCI-H1581, NCI-H716, NCI-H2444 and Hep3B cells were obtained from the American Type Culture Collection (USA). RT112 cell was obtained from Deutsche Smmlung von Mikroorganismen und Zellkulturen GmbH (Germany). All cell lines in this study were maintained in the appropriate medium as suppliers suggested and were authenticated via short tandem repeats (STR) analysis with the latest test in 2020 (Genesky Biotechnologies, China) or single-nucleotide polymorphism (SNP) analysis with the latest test in 2021 (Crown Bioscience, China). All cells were routinely tested for mycoplasma by Mycoplasma Detection Kit-QuickTest (B39032; Biotool, China) and found to be free of contamination.

FGFR inhibitors (Erdafitinib, AZD4547, and BLU9931), AKT inhibitor MK2206, mTOR inhibitor AZD8055, proteasome inhibitor MG132 and lysosome inhibitor Leupeptin were purchased from Selleck Chemicals (China) and dissolved in DMSO at the concentration of 10 mM as a stock solution for *in vitro* study. AZD4547 was dissolved in 1% Tween-80 and BLU9931 was formulated in 0.5% carboxymethylcellulose/1% Tween-80 respectively for *in vivo* study.

To generate NCI-H1581 cells with acquired resistance to FGFRi, NCI-H1581 cells were treated by AZD4547 with the increasing concentration in a stepwise manner (from 30 nM to 1 μM). After approximate 6 months of induction, the NCI-H1581/AR cell line was obtained till its growth kinetics was similar to that of the parental NCI-H1581 cell line 33.

### Mass spectrum

Protein extraction, digestion, Tandem Mass Tag (TMT) labeling and high pH reversed-phase liquid chromatography peptides fractionation were performed as described previously 34. Detailed methods were available in the Supplementary Materials. The mass spectrometry proteomics data have been deposited to the ProteomeXchange Consortium via the PRIDE 35 partner repository with the dataset identifier PXD032227.

### ^18^F-FDG uptake *in vitro*

For adherent cells, 2.0×10^5^ cells/well were seeded in 12-well plates and 12 h FGFRi treatment at indicated concentrations in normal medium was started next day, followed by another 12 h FGFRi treatment in the glucose-free starvation medium with 5% fetal bovine serum. For non-adherent cells, 3.0×10^5^ cells/well were seeded in 12-well plates in normal medium with FGFRi at indicated concentrations for 12 h, followed by another 12 h FGFRi treatment in the above starvation medium. After starvation, 1 µCi ^18^F-FDG/well was then added and incubated at 37°C for 1 h. The radioactivity from both cell-accumulated and free ^18^F-FDG were measured by an automatic gamma counter (Wizard 2470; PerkinElmer, USA). Relative ^18^F-FDG uptake rate was normalized by cell number analyzed using Countstar BioTech (China). All samples were tested in triplicate.

### Cell viability assay

Cells were inoculated in 96-well plates overnight and incubated with FGFRi at indicated concentrations or vehicle (as a negative control) for 72 h. Cell Counting Kit-8 (Dojindo Molecular Technologies, China) was used to assess cell viability as the instruction described. The normalized cell viability (%) was calculated as 100 × (OD_FGFRi_/OD_vehicle_).

### Western blot analysis

Total cellular protein was extracted by 1×SDS lysis buffer and denatured at 100℃ for 15 min. Then the protein samples were loaded in 10% or 12.5% SDS-PAGE and transferred to a nitrocellulose membrane. After 1 h blocking with 3% BSA (Sigma-Aldrich, USA) at room temperature, the membrane was incubated with the primary antibodies from Cell Signaling Technology (USA): p-FGFR (Y653/654, #3476; 1:1,000), p-FRS2 (Y436, #3861; 1:500), p-ERK (T202/Y204, #4370; 1:1,000), p-AKT (S473, #4060; 1:1,000), p-p70S6K (T421/S424, #9204; 1:1,000), p-4EBP1 (T70, #9455; 1:1,000), HK1 (#2024; 1:1,000), HK2 (#2867; 1:1,000), GLUT1 (#12939; 1:1,000), PLCγ (#5690; 1:1,000), ERK (#4695; 1:1,000), AKT (#4691; 1:1,000), p70S6K (#2708; 1:1,000), 4EBP1 (#9644;1:1,000), β3-Tubulin (#5666; 1:1,000), and β-Actin (#3700; 1:10,000), or from Abcam (USA): GLUT3 (#ab41525; 1:1,000), and c-Myc (#ab12939; 1:1,000), or from Millipore (USA): p-PLCγ (Y783, #07-2134; 1:1,000), or from Kangcheng Bio (China): GAPDH (#KC-5G4; 1:20,000) at 4℃ overnight respectively. The membrane was then incubated with the corresponding secondary antibodies from Jackson ImmunoResearch (USA) (HRP-conjugated anti-rabbit IgG (#111-035-003; 1:2,000) and HRP-conjugated anti-mouse IgG (#115-035-003; 1:2,000)) at room temperature for 1 h, respectively. Clarity Western ECL Substrate (Bio-Rad, USA) was used to visualize the blots and images were captured by ImageQuant LAS-4000 imager (GE Healthcare, USA). Western blots were quantified using Image J software and relative band intensity of target protein was normalized to its corresponding loading control as fold of the vehicle-, or control-, or non-treated group.

### Gene silencing by siRNA

Cells were inoculated in 6-well plates overnight and transfected with siRNAs as below by Oligofectamine RNAiMAX reagent (Invitrogen, USA) according to the manufacturer's instructions. After 48 h, cells were harvested for further analysis.

*PLCG1* siRNA-1: 5'-AAGAAGUCGCAGCGACCCGAG-3'

*PLCG1* siRNA-2: 5'-GGGACUUUGAUCGCUAUCATT-3'

*MYC* siRNA-1: ON-Target plus SMARTpool Human MYC (L-003282-02; Dharmacon, USA)

*MYC* siRNA-2: 5'-GGACUAUCCUGCUGCCAAGTT-3'

### Quantitative real-time PCR

Total RNA was extracted using TRIzol reagent (Invitrogen) and subjected to reverse transcription with 5×HiScript II qRT SuperMix II (Vazyme, China). PCR was performed with 2×ChamQ Universal SYBR qPCR Master Mix (Vazyme). Primers for HK2 mRNA were as below:

*HK2* forward: 5'-GAGCCACCACTCACCCTACT-3'

*HK2* backward: 5'-CCAGGCATTCGGCAATGTG-3'

### Animal studies

All animal studies were approved by the Institutional Animal Care & Use Committee of Shanghai Institute of Materia Medica, Chinese Academy of Sciences. 4- to 6-week-old female athymic nude mice nu/nu or SCID mice were provided by Shanghai Institute of Materia Medica or purchased from Beijing HFK Bioscience (China). 1×10^7^ tumor cells, including NCI-H1581, Hep3B, NCI-H2444, or NCI-H1581/AR cells, were suspended in 200 μl ice-cold sterile PBS and subcutaneously injected into right flank of the mouse. Tumor-bearing mice were divided into the vehicle group and FGFRi treatment group randomly when the tumor volume reached approximately 100 mm^3^. For NCI-H1581, NCI-H1581/AR, and NCI-H2444 xenograft-bearing mice, AZD4547 (12.5 mg/kg, p.o., once a day) was given for 4 or 5 days. For Hep3B xenograft-bearing mice, BLU9931 (30 mg/kg, p.o., twice a day) was given for 6 days. Tumor size was measured by caliper every day and tumor volume (TV) was calculated with the formula: TV = (width^2^ × length) / 2. The relative tumor volume was normalized by the TV immediately before FGFRi treatment.

### PET/CT imaging

The tumor-bearing mice were fasted for 8 h before injection of 100-200 µCi ^18^F-FDG via tail vein. During the uptake period (40-60 min), the mice were anesthetized under 1.5% isoflurane. Ten-min static data of PET imaging were recorded, followed by 10-min CT scan, using a microPET/CT scanner (Inveon; Siemens, Germany). PET data were reconstructed using the microQ Viewer software (Version 1.7.0.6; Siemens). Region of interest (ROI) delineating the tumor was drawn and Mean Standardized Uptake Value (SUVmean) of the tumor was obtained for ^18^F-FDG uptake *in vivo*.

### Immunohistochemistry (IHC)

Tumor tissues were fixed with 4% paraformaldehyde for at least 12 h, followed by dehydration and paraffin embedding. Five µm-thick paraffin sections were cut and antigen was retrieved by boiling in citrate buffer (pH 6.0) for 30 min. Primary antibodies against HK2 (#2867; Cell Signaling Technologies; 1:200) or Ki67 (#9027; Cell Signaling Technologies; 1:400), and UltraSensitive SP (for rabbit) IHC Kit (KIT-9707; Maixin_Bio, China) were used sequentially. The slides were stained with a DAB visualization kit (DAB-0031; Maixin_Bio) and counterstained with hematoxylin. IHC analysis for HK2 was performed by Zuocheng Biotech (China). Images were captured by a slide scanner (NanoZoomer 2.0-HT; Hamamatsu, Japan).

### Statistical analysis

Data were presented as mean ± SD, except the data for xenograft growth curve, which were presented as mean ± SEM. The differences between two groups were analyzed by an unpaired Student's t-test using GraphPad Prism 8.0 software (USA). FGFR4-HK2 signature score was determined by Cox model 36. Briefly, this score of each patient was calculated as follows: FGFR4-HK2 signature score = X_FGFR4_β_FGFR4_ + X_HK2_β_HK2_ (X and β indicated the mRNA level and the risk coefficient of Cox model by survival analysis in R version 3.5.3, respectively). The correlations between the levels of FGFR4 mRNA/HK2 mRNA/FGFR4-HK2 signature score and overall survival (OS) were analyzed by the Kaplan-Meier method. The differences in the survival rates between curves were assessed by the log-rank test. p < 0.05 was considered statistically significant.

## Results

### FGFR inhibition led to ^18^F-FDG uptake reduction in the FGFRi-sensitive cancer cells

**Figure 1:**
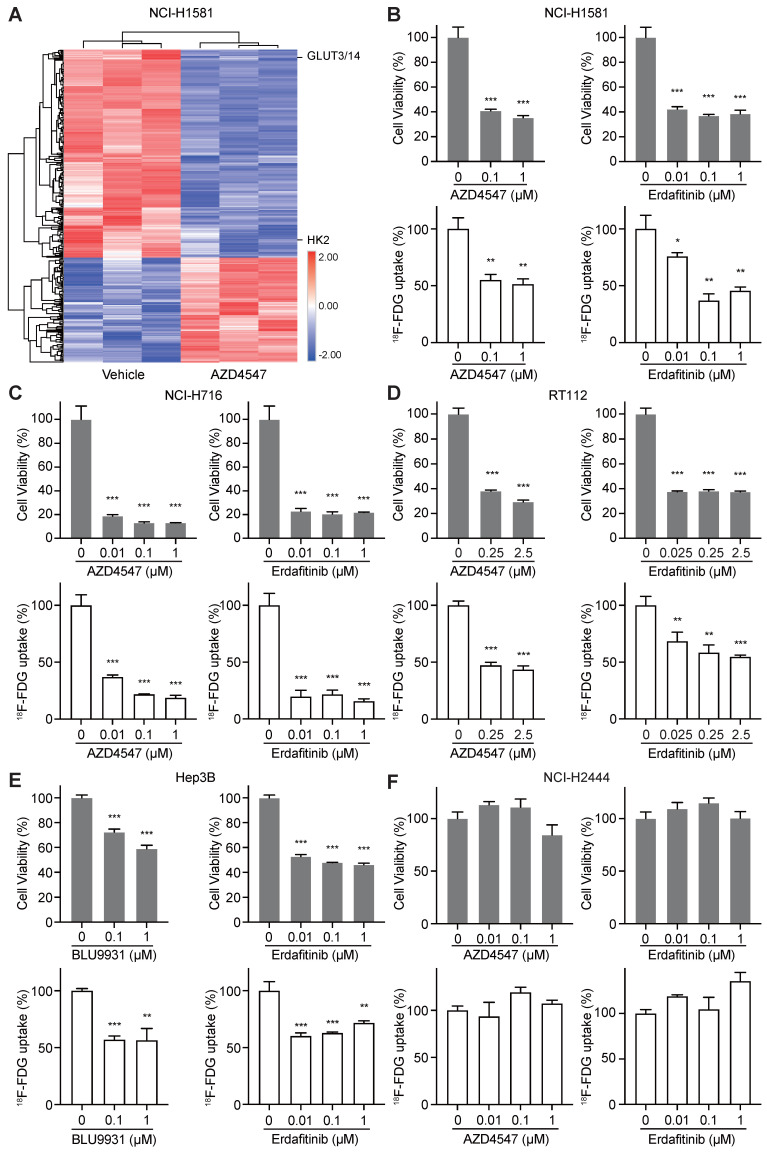
** FGFR inhibition resulted in ^18^F-FDG uptake reduction in FGFRi-sensitive cancer cells. A**, NCI-H1581cells were treated with AZD4547 (0.1 µM) or vehicle for 24 h, then protein lysates were collected for TMT-labeled mass spectrometry-based proteomics analysis. Hierarchical clustering analysis was performed using the differentially expressed proteins upon AZD4547 treatment (fold change > 1.2 or < 0.8, p < 0.05, Z-score transformed). **B-F**, NCI-H1581 (**B**), NCI-H716 (**C**), RT112 (**D**), Hep3B (**E**) and NCI-H2444 (**F**) cells were incubated with indicated FGFR inhibitors (AZD4547, Erdafitinib, or BLU9931) at different concentrations. Cell viability (upper panels) and ^18^F-FDG uptake (lower panels) were examined after 72 h and 24 h, respectively. Cells treated with vehicle were used as the normalization controls. Relative ^18^F-FDG uptake was normalized by cell number. Data were shown as mean ± SD. *, p < 0.05; **, p < 0.01; ***, p < 0.001 vs vehicle group, using Student's t-test.

To identify novel biomarkers for evaluating the therapeutic response to FGFRi, TMT-labeled mass spectrometry-based proteomics was carried out in a FGFRi-sensitive cell line (NCI-H1581, a lung cancer cell line with FGFR1 amplification) upon AZD4547 treatment (Figure 1A). As expected, proteins associated with cell cycle regulation were identified among the significantly differentially expressed ones (fold change > 1.2 or < 0.8, with p < 0.05; Table S1), which was consistent with our previous report 21. Notably, protein levels of HK2 and GLUT3/14 (alias SLC2A3/14), the key factors highly related to glucose metabolism, especially to FDG trapping, were significantly decreased in AZD4547-treated NCI-H1581 cells than those in vehicle-treated ones (Figure 1A). Therefore, ^18^F-FDG uptake was tested in NCI-H1581 cells with FGFRi treatment *in vitro*. Two selective inhibitors, AZD4547 targeting FGFR1-3 and Erdafitinib targeting FGFR1-4 were used. We observed that both AZD4547 (0.1 μM) and Erdafitinib (0.01 μM) not only inhibited the cell proliferation (p < 0.001) but also reduced the ^18^F-FDG uptake (p < 0.05) (Figure 1B).

Whether FGFR inhibition leading to ^18^F-FDG uptake reduction is a common effect on FGFR-aberrant tumor cells was further investigated. Four other cancer cell lines, the FGFRi-sensitive ones including NCI-H716 colon cancer cell line with FGFR2 amplification, RT112 bladder cancer cell line with FGFR3 amplification, and Hep3B liver cancer cell line with FGF19 amplification-induced FGFR4 activation, along with the FGFRi-primary resistant one (NCI-H2444 lung cancer cell line with FGFR1 amplification), were examined. Another FGFR4 selective inhibitor, BLU9931 was chosen for tests in Hep3B cells. Consistently, FGFRi-induced significant cell proliferation inhibition was still accompanied with ^18^F-FDG uptake decrease in NCI-H716 cells (p < 0.001; Figure 1C), RT112 cells (p < 0.01; Figure 1D), and Hep3B cells (p < 0.01; Figure 1E). However, in NCI-H2444 cells both AZD4547 and Erdafitinib did not show the inhibitory effects on cell proliferation and ^18^F-FDG uptake even at the concentration of 1 μM (Figure 1F). These data implied that ^18^F-FDG uptake might be correlated with the drug sensitivity/resistance to FGFRi in FGFR-aberrant cancer cells.

### FGFR inhibition downregulated *HK2* gene via mTOR pathway

**Figure 2:**
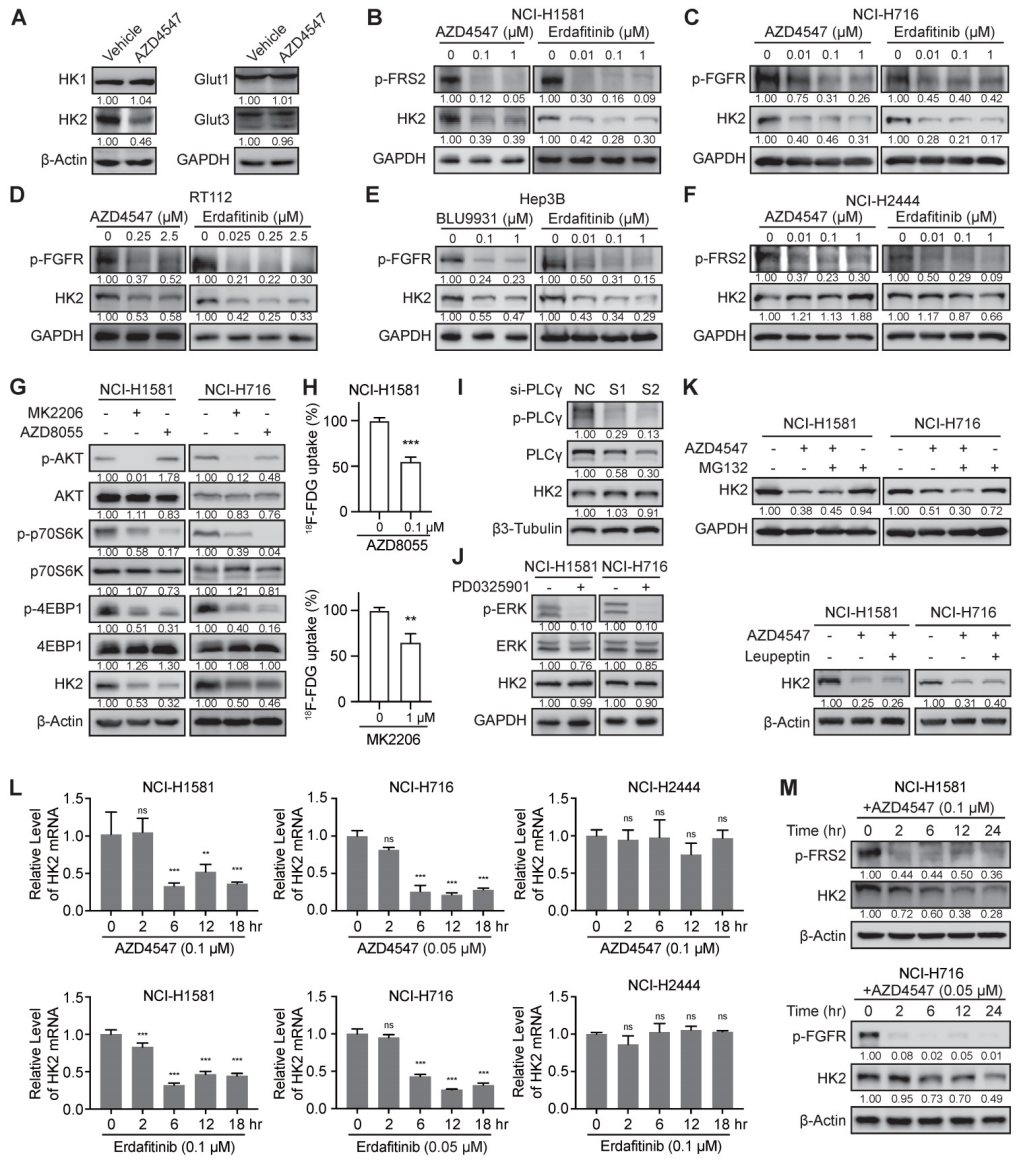
** FGFR inhibition suppressed the transcription level of *HK2* gene via mTOR pathway. A**, Western blot analysis on glucose metabolism-related proteins in NCI-H1581 cells with AZD4547 treatment (0.1 µM) for 24 h. β-Actin and GAPDH were used as the loading controls. **B-F**, Western blot analysis on protein levels of HK2 and FGFR signalings (p-FGFR or p-FRS2) with FGFR inhibitors treatment as indicated for 24 h in NCI-H1581 (**B**), NCI-H716 (**C**), RT112 (**D**), Hep3B (**E**) and NCI-H2444 (**F**) cells. GAPDH was used as the loading control. **G**, Western blot analysis on protein levels of HK2 and AKT/mTOR signalings with AKT inhibitor MK2206 (1 μM) or mTOR inhibitor AZD8055 (0.1 μM) for 24 h in NCI-H1581 and NCI-H716 cells. β-Actin was used as the loading control. **H**, ^18^F-FDG uptake by NCI-H1581 cells treated with AZD8055 or MK2206 for 24 h. **I**, Western blot analysis on protein levels of HK2 and PLCγ signalings in NCI-H1581 cells transiently transfected with two siRNAs targeting PLCγ (S1 and S2) or siRNA control (NC) for 48 h. β3-Tubulin was used as the loading control. **J**, Western blot analysis on protein levels of HK2 and MEK signalings with MEK1/2 inhibitor PD0325901 (1 μM) for 24 h in NCI-H1581 and NCI-H716 cells. GAPDH was used as the loading control. **K**, Western blot analysis on HK2 protein levels in NCI-H1581 and NCI-H716 cells with AZD4547 treatment (0.1 μM for NCI-H1581 cells, and 0.05 μM for NCI-H716 cells) for 24 h. MG132 (10 μM, upper panel) or Leupeptin (10 μM, lower panel) was added 6 h before sample collection. β-Actin and GAPDH were used as the loading controls. **L**, Quantitative RT-PCR analysis for mRNA levels of *HK2* gene at the indicated time points in NCI-H1581 (left panel), NCI-H716 (middle panel) and NCI-H2444 cells (right panel) with AZD4547 or Erdafitinib treatment. Cells without treatment (0 h) were used as the normalization controls. **M**, Western blot analysis on protein levels of HK2 and FGFR signalings at the indicated time points in NCI-H1581 (upper panel) and NCI-H716 cells (lower panel) with AZD4547 treatment. β-Actin was used as the loading control. Data were shown as mean ± SD. **, p < 0.01; ***, p < 0.001; ns, p ≥ 0.05. Relative band intensity of target protein was normalized to its corresponding loading control as fold of the vehicle-, or control-, or non-treated group.

Since mass spectrometry-based proteomics identified that AZD4547 could decrease the protein levels of HK2 and GLUT3/14 (Figure 1A), which are the main mediators of ^18^F-FDG uptake, we tested the expression levels of these two molecules and other members in HK and GLUT families by Western blot analysis in NCI-H1581 cells for confirmation (Figure 2A). Due to the subtle change of GLUT3 and the specific expression of GLUT14 major in testis 37, only the significant inhibitory effects of FGFRi on HK2 expression levels were further investigated. Three FGFR inhibitors (Erdafitinib, AZD4547 or BLU9931) were tested correspondingly in five FGFR-aberrant cancer cells. In the FGFRi-sensitive cells, including NCI-H1581 (Figure 2B), NCI-H716 (Figure 2C), RT112 (Figure 2D), and Hep3B cells (Figure 2E), FGFRi reduced the phosphorylated levels of FGFR (p-FGFR) or FRS2 (p-FRS2), which is the FGFR key adaptor protein as the well-recognized surrogate for FGFR activation 10,11,21,38, as well as decreased the protein levels of HK2. But in the FGFRi-resistant cells (NCI-H2444 cells), even p-FRS2 was suppressed by AZD4547 or Erdafitinib treatment, HK2 protein did not show significant changes (Figure 2F). It was suggested that FGFRi could inhibit HK2 only in the FGFRi-sensitive cells.

How FGFR inhibition downregulated HK2 was then studied. Considering the FGFR aberration usually activates AKT-mTOR, PLCγ, and MEK-ERK pathways in cancer 1,2, we used the selective inhibitors or specific siRNAs to block these downstream signalings to test which could downregulate HK2. As Figure 2G-H shown, both the AKT inhibitor (MK2206) and the mTOR inhibitor (AZD8055) reduced the HK2 levels and ^18^F-FDG uptake, as same as the FGFRi. However, neither knockdown PLCγ by siRNAs in NCI-H1581 cells (Figure 2I) nor MEK inhibition by PD0325901 in NCI-H1581 and NCI-H716 cells (Figure 2J) affected the HK2 levels. We also knockdown c-Myc, which was the downstream effector of FGFR via MEK-ERK signaling in FGFR aberrant cancer 21, and no obvious HK2 expression change was exhibited in NCI-H1581 and Hep3B cells (Figure S1), indicating the different regulatory mechanisms by FGF/FGFR for HK2 and c-Myc. Herein, function of FGFRi's tumor inhibition may not be actioned simply by FGFR pathway; FGFR inhibition induced HK2 reduction via AKT-mTOR signaling to regulate glucose metabolism was indicated in the FGFRi-sensitive cells.

Whether FGFR inhibition downregulated HK2 expression at posttranslational level was next addressed. Neither the proteasome inhibitor MG132 nor the lysosome inhibitor Leupeptin could reverse HK2 downregulation induced by FGFR inhibition in NCI-H1581 (Figure 2K), NCI-H716 (Figure 2K) and Hep3B cells (Figure S2), suggesting this downregulation was not greatly dependent on protein degradation. mRNA levels of *HK2* gene in NCI-H1581, NCI-H716, and NCI-H2444 cells were then detected at different time points (0-18 h after FGFRi treatment).

The significant decrease of HK2 mRNA was observed in FGFRi-sensitive cells starting from ~6 h after FGFR inhibition (Figure 2L). Accordingly, HK2 protein level exhibited a slight reduction beginning from 6 h after FGFRi treatment and achieved a significant decrease after 12- or 24-h treatment in those cells (Figure 2M). In FGFRi-resistant NCI-H2444 cells, no significant change of HK2 mRNA was detected under the treatment of AZD4547 or Erdafitinib (Figure 2L). Furthermore, mTOR inhibitor (AZD8055) decreased expressional levels of HK2 mRNA (Figure S3A) and HK2 protein in both FGFRi-sensitive (Figure 2G) and -resistant cells (Figure S3B). However, the mTOR signaling (Figure S3C) and HK2 protein (Figure 2F) did not show significant changes by AZD4547 or Erdafitinib treatment in NCI-H2444 cells, implying that FGFR might lose its regulation on mTOR signaling, but mTOR could still modulate the expression of HK2 gene in this FGFR-resistant cell line.

The correlation between FGFR/HK2 signaling and liver cancer patients' prognosis was tested in TCGA-LIHC (liver hepatocellular carcinoma) dataset. The patients with OS information (n = 373) were classified into two groups based on the levels of FGFR4 mRNA, HK2 mRNA and FGFR4-HK2 signature score, respectively (median value as the cutoff). The Kaplan-Meier survival analysis showed that high levels of FGFR4 mRNA (p = 0.0232), HK2 mRNA (p = 0.0057) and FGFR4-HK2 signature score (p = 0.0027) were associated with poor OS of LIHC patients (Figure S4). The importance of FGFR/HK2 signaling in the prognosis of LIHC patients was indicated.

Taken together, above results suggested that FGFR inhibition downregulated *HK2* gene transcription via AKT-mTOR signaling, leading to the decrease of glucose uptake in the FGFRi-sensitive tumor cells.

### ^18^F-FDG PET as an imaging biomarker for the therapeutic response to FGFRi *in vivo*

**Figure 3:**
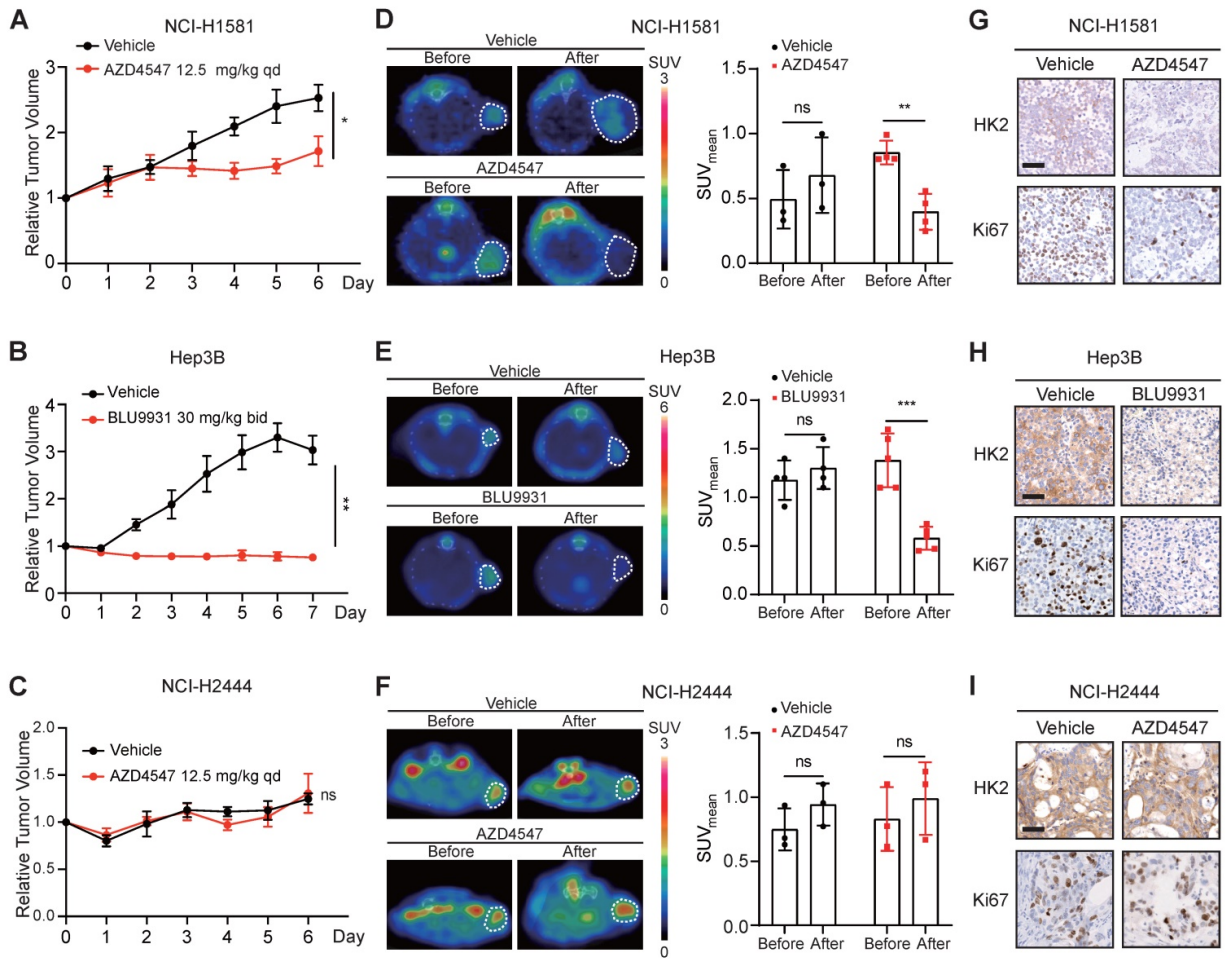
** Tumor ^18^F-FDG uptake in the xenograft-bearing mice upon FGFRi treatment by PET/CT imaging.** Nude mice bearing NCI-H1581 or NCI-H2444 xenografts were orally treated with AZD4547 at 12.5 mg/kg or vehicle daily for 5 days. SCID mice bearing Hep3B xenografts were orally treated with BLU9931 at 30 mg/kg or vehicle twice a day for 6 days. **A-C**, Relative tumor growth curves of NCI-H1581 xenografts (**A**, n = 3 for vehicle group, n = 4 for AZD4547 group), Hep3B xenografts (**B**, n = 4 for vehicle group, n = 5 for BLU9931 group) and NCI-H2444 xenograft (**C**, n = 3 for each group) within the indicated periods. Xenograft volumes at the starting point (0 day) were used as the normalization controls. Data were shown as mean ± SEM. *, p < 0.05; **, p < 0.01; ns, p ≥ 0.05 vs vehicle group, using Student's t-test. **D-F**, ^18^F-FDG PET/CT imaging on the NCI-H1581 (**D**), Hep3B (**E**) and NCI-2444 (**F**) xenograft-bearing animals upon FGFRi or vehicle treatment. Left panels: representative ^18^F-FDG PET/CT images (xenografts were indicated with white dashed lines). Right panels: mean standardized uptake values (SUVmean) from the xenografts before and after FGFRi or vehicle treatment; data were shown as mean ± SD. **, p < 0.01; ***, p < 0.001; ns, p ≥ 0.05. **G-I**, IHC staining for HK2 and Ki67 of NCI-H1581 (**G**), Hep3B (**H**) and NCI-2444 xenografts (**I**) after FGFRi or vehicle treatment. Scale bar, 50 μm.

In order to investigate whether ^18^F-FDG PET could be used as an imaging biomarker for the therapeutic response to FGFRi *in vivo*, the FGFRi-sensitive xenografts, NCI-H1581 (Figure 3A) and Hep3B (Figure 3B), as well as the FGFRi-primary resistant xenografts (NCI-H2444; Figure 3C) were generated for FGFRi treatment with visualization by ^18^F-FDG PET/CT imaging *in vivo*. NCI-H1581 and NCI-H2444 xenograft-bearing mice were treated with AZD4547 (12.5 mg/kg, daily) for 5 days; and Hep3B xenograft-bearing mice were treated with BLU9931 (30 mg/kg, twice a day) for 6 days. Upon FGFRi treatment, SUVmean for ^18^F-FDG probe was significantly reduced in NCI-H1581 xenografts (p < 0.01; Figure 3D) and in Hep3B xenografts (p < 0.001; Figure 3E); meanwhile, vehicle could not induce such decrease. In NCI-H2444 xenografts, marked change of ^18^F-FDG probe was not detected in both AZD4547- and vehicle-treatment groups (Figure 3F). The following IHC analysis confirmed that FGFRi treatment led to the reductions of HK2 and Ki67 (a cell proliferation marker) only in the FGFRi-sensitive xenografts (Figure 3G-H), but not in the FGFRi-resistant xenografts (Figure 3I).

**Figure 4:**
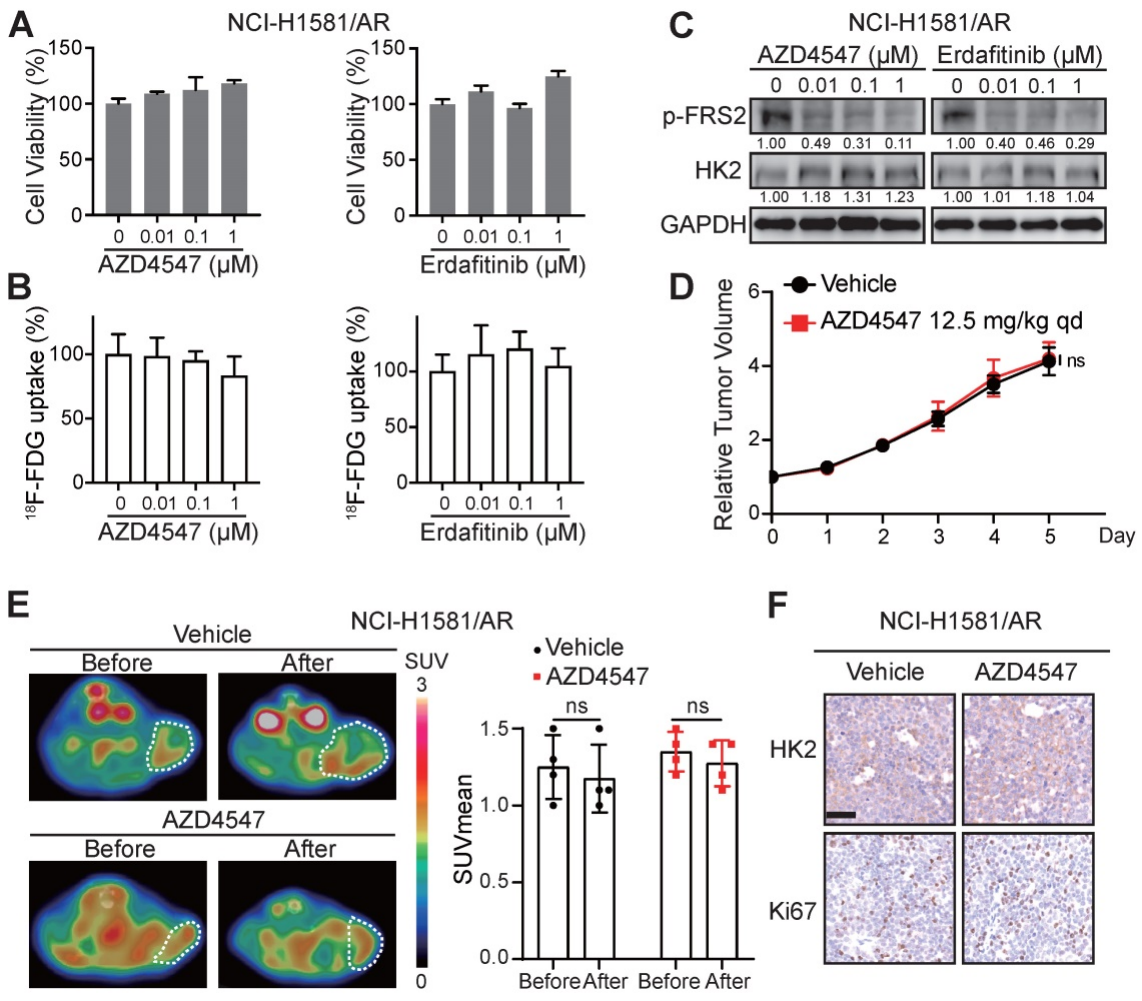
** Loss of ^18^F-FDG uptake reduction to FGFR inhibitors in NCI-H1581/AR tumor cells* in vitro* and *in vivo*. A-B**, NCI-H1581/AR cells were incubated with indicated FGFR inhibitors (AZD4547 and Erdafitinib) at different concentrations. Cell viability (**A**) and ^18^F-FDG uptake (**B**) were examined after 72 h and 24 h, respectively. Cells treated with vehicle were used as the normalization controls. Data were shown as mean ± SD. **C**, Western blot analysis on protein levels of HK2 and FGFR signaling (p-FRS2) with FGFR inhibitors treatment as indicated for 24 h in NCI-H1581/AR cells. GAPDH was used as the loading control. **D**, SCID mice bearing NCI-H1581/AR xenografts were orally treated with AZD4547 at 12.5 mg/kg or vehicle daily for 4 days. Relative tumor growth curves of NCI-H1581/AR xenografts (n = 4 for each group) within the indicated periods. Xenograft volumes at the starting point (0 day) were used as the normalization controls. Data were shown as mean ± SEM. ns, p ≥ 0.05 vs vehicle group, using Student's t-test. **E**, ^18^F-FDG PET/CT imaging on the NCI-H1581/AR xenograft-bearing animals upon FGFRi or vehicle treatment. Left panel: representative ^18^F-FDG PET/CT images (xenografts were indicated with white dashed lines). Right panel: SUVmean from the xenografts before and after FGFRi or vehicle treatment; data were shown as mean ± SD. ns, p ≥ 0.05. **F**, IHC staining for HK2 and Ki67 of NCI-H1581/AR xenografts after FGFRi or vehicle treatment. Scale bar, 50 μm. Relative band intensity of target protein was normalized to its corresponding loading control as fold of the vehicle-treated group.

These data encouraged us to explore whether ^18^F-FDG uptake in the FGFRi-acquired resistant tumors would be similar to that in the FGFRi-primary resistant tumors. NCI-H1581/AR cell line, which was previously generated from NCI-H1581 parental cell line by exposure to AZD4547 at concentrations increasing stepwise 33, was tested *in vitro* and *in vivo*. Its resistance to AZD4547 and Erdafitinib was validated (Figure 4A). Consistent with the results from NCI-H2444 cells, ^18^F-FDG uptake (Figure 4B) and HK2 protein level (Figure 4C) in NCI-H1581/AR cells did not show the remarkable changes in presence of AZD4547 or Erdafitinib. NCI-H1581/AR subcutaneous xenograft model was then created. Using the same dosage of AZD4547 as that in NCI-H1581 xenograft-bearing mice for 4 days, tumor growth could not be inhibited in NCI-H1581/AR xenografts (Figure 4D). ^18^F-FDG-based PET/CT imaging could not detect significant alterations of the probe accumulation in NCI-H1581/AR xenografts, responding to AZD4547 treatment (Figure 4E). FGFRi treatment *in vivo* could not alter the protein levels of HK2 and Ki67 in NCI-H1581/AR xenografts (Figure 4F). It was implied that the acquired resistance to targeted FGFR therapy might accompany by the disability of FGFRi-induced FDG uptake reduction.

### Application of ^18^F-FDG PET imaging to monitor the therapeutic response to FGFRi *in vivo* dynamically

**Figure 5:**
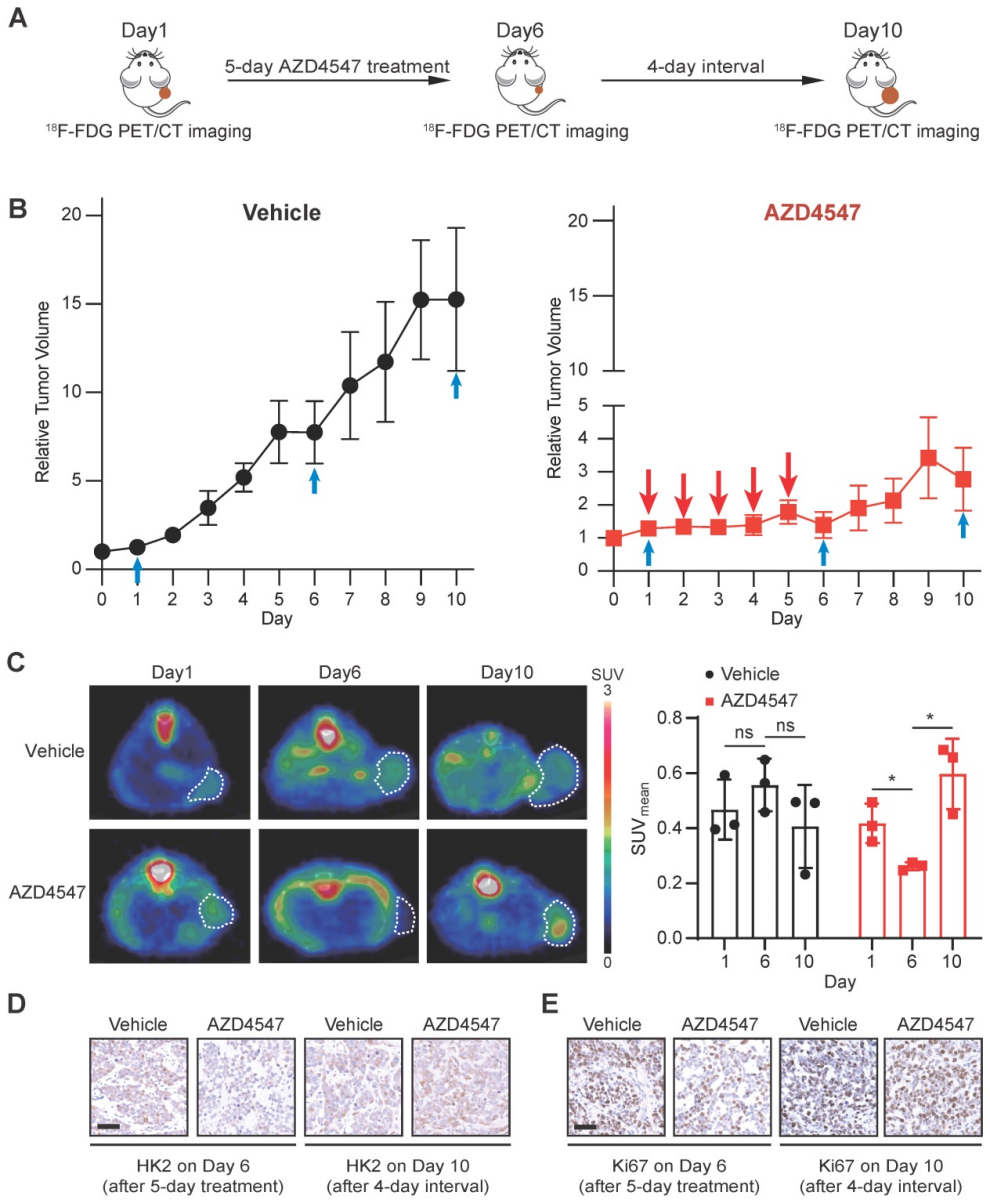
**^18^F-FDG PET imaging for monitoring the therapeutic response to FGFRi *in vivo* dynamically. A**, Schematic diagram illustrating the experimental design. Nude mice bearing NCI-H1581 xenograft were orally treated with AZD4547 at 12.5 mg/kg once a day for 5 days, then AZD4547 was withdrawn for 4 days. ^18^F-FDG PET/CT imaging was performed on Day 1 (before treatment), Day 6 (right after treatment) and Day 10 (after treatment interval). **B**, Relative tumor growth curves of NCI-H1581 xenografts (n = 3 for each group) within the indicated periods. Blue arrows, time points with ^18^F-FDG PET/CT imaging; red arrows, time points with AZD4547 treatment. Xenograft volumes at the starting point (0 day) were used as the normalization controls. Data were shown as mean ± SEM. **C**, ^18^F-FDG PET/CT imaging on the NCI-H1581 xenograft-bearing animals upon FGFRi or vehicle treatment. Representative ^18^F-FDG PET/CT images for SUVmean (left panel; xenografts were indicated with white dashed lines) and values for SUVmean from the xenografts (right panel) at the indicated time points were shown. Data were shown as mean ± SD. ns, p ≥ 0.05; *, p < 0.05. **D-E**, IHC staining for HK2 (**D**) and Ki67 (**E**) of NCI-H1581 xenografts on Day 6 (right after treatment) and Day 10 (after treatment interval). Scale bar, 50 μm.

To mimic the clinical practice of targeted FGFR therapy, we generated a NCI-H1581 xenograft model with the treatment regimen as a 5-day FGFRi treatment followed by a 4-day interval. ^18^F-FDG PET/CT imaging was applied to assess the glucose uptake at three time points: 1) right before FGFRi treatment (Day 1); 2) right after FGFRi treatment (Day 6); 3) at the endpoint (Day 10; 4 days without FGFRi treatment) (Figure 5A). Using this model, we wanted to investigate whether ^18^F-FDG PET could reflect the tumor response to FGFRi in a dynamic and quantitative manner.

The tumor growth curves measured by calipers (Figure 5B) showed that in the vehicle group, tumors kept growing with ~13.6-fold increase in tumor volume; in the AZD4547 group, tumor growth was significantly inhibited within the first phase (5-day with AZD4547), whereas tumor volume was slightly increased with ~1.9-fold increase in the second phase (4-day without AZD4547). As shown in Figure 5C, ^18^F-FDG PET images demonstrated that vehicle treatment did not induced obvious changes in ^18^F-FDG uptake by the tumor; and that 5-day FGFRi treatment resulted in a marked decrease of ^18^F-FDG uptake by the tumor (p < 0.05). Notably, 4-day FGFRi withdrawal led to more ^18^F-FDG accumulation in tumor (~2.3-fold increase in SUVmean on Day 10 compared with that on Day 6; p < 0.05), as well as the increased HK2 (Figure 5D) and Ki67 levels (Figure 5E). ^18^F-FDG PET enabled the assessment of FGFRi therapeutic efficacy dynamically *in vivo* was indicated.

## Discussion

FGFRs are clinically validated anticancer targets with pan-tumor potential, especially in the tumors lacking effective treatments. However, the clinical benefit in cancer patients with FGFR alterations is quite limited 39-41. Moreover, even FGFR-aberrant patients attain an optimal response at an early stage, tumor relapse occurs eventually due to acquired resistance by the activation of bypass and downstream signalings or the development of FGFR secondary mutations 1. Biomarkers or strategies with immediate translational potentials to evaluate the therapeutic response and to monitor the acquired resistance to FGFR-targeted therapy are urgently needed, particularly in a noninvasive and dynamic manner.

**Figure 6:**
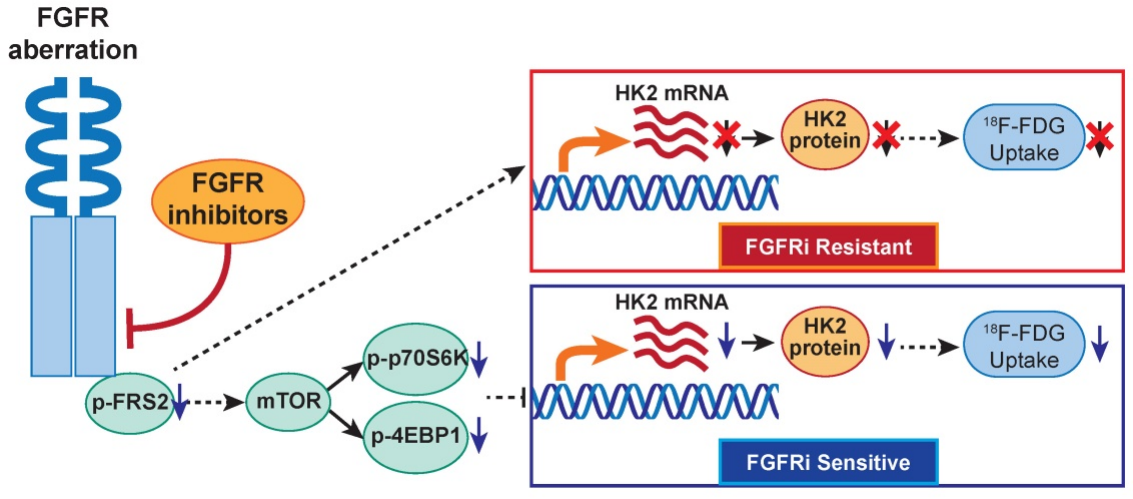
**The proposed working model.** In FGFR-aberrant cancers, the therapeutic response of FGFR inhibitors visualized by ^18^F-FDG PET/CT imaging is correlated with FGF/FGFR signaling-mediated glucose metabolism via mTOR/HK2 axis.

In the present study, TMT-labeled mass spectrometry-based proteomics suggested that FGFR inhibition regulated glucose metabolism in a FGFRi-sensitive cancer cell line NCI-H1581 with FGFR1 amplification. Interestingly, our previous report showed that cancer cells with FGFR-aberrant activation *per se* exhibited high glucose consumption into glycolytic pathway and resultant lactate production 42. The critical role of glucose metabolism in FGFR-aberrant cancers is indicated, no matter with FGFRi treatment or not. In TCGA lung adenocarcinoma database (n = 740), upregulation of some glycolytic enzymes including *HK2* gene was reported in FGFR-amplificated cancers, comparing with diploid cancers 42. These findings encouraged us to further investigate whether FGFR kinase-targeted therapy was able to regulate HK2 expression and thereby inhibit glycolysis herein. Mechanistically, for the first time, we revealed that FGFR inhibition suppressed *HK2* gene transcription via inhibiting mTOR in FGFRi-sensitive *in vitro* and *in vivo* cancer models with different FGFR1-4 anomalies (Figure 6). In the FGFRi-sensitive cells, we found only mTOR inhibition could suppress HK2 expression; while inhibitions of other FGFR key downstream molecules (PLCγ and MEK/ERK) did not show the same inhibitory effect on HK2. Considering that c-Myc functioned as a key downstream effector in aberrantly activated FGFR signaling in cancer 21 and that FGF-induced vascular development was dependent on endothelial glycolysis via MYC/HK2 43, we also tested whether HK2 downregulation by FGFRi was c-Myc dependent in this study. However, we found c-Myc knockdown had nonsignificant influence on HK2 expression level. Additionally, since HK2 can be regulated by several factors including epigenetic factors 44, perhaps HK2 expression might serve as a biomarker independently of FGFR aberration. Such different regulatory mechanisms on HK2 may partially be owing to the differences in biological context and cell lineage. The novel FGFR/mTOR/HK2 axis-mediated glucose metabolic regulation to assess the response to FGFR-targeted therapy is suggested.

The disturbed glucose metabolism, as metabolic vulnerability for FGFR-addicted cancers, especially the corresponding expressional change of HK2, the key rate-limiting glycolytic enzyme, allows us trying to monitor the FGFRi-response by ^18^F-FDG (an analog of glucose) PET imaging. We demonstrated that FGFR inhibition reduced ^18^F-FDG *in vitro* uptake in four FGFRi-sensitive cancer cells, but not in primary and acquired resistant cancer cell lines to FGFRi. ^18^F-FDG PET/CT imaging and IHC analysis on the tumor xenograft-bearing animals further confirmed the *in vivo* decreases of HK2 expression, ^18^F-FDG tumor accumulation and tumor proliferation upon FGFRi treatment only in FGFRi-sensitive tumors; whereas these decreases were not observed in the FGFRi- de novo and acquired resistant tumors. The change of ^18^F-FDG tumor uptake at early treatment stage might be used to identify the primary FGFRi-resistant patients noninvasively, despite the presence of FGFR activating mutations, to avoid unnecessary ineffective treatment and spare costs. Furthermore, both ^18^F-FDG tumor accumulation and HK2 expression could respond the administration/withdrawal of FGFRi in NCI-H1581 xenografts correspondingly, which in turn suggested that the loss of ^18^F-FDG tumor uptake response to FGFRi treatment might be associated with the acquired FGFRi-resistance. Certainly, using an inducible HK2 xenograft model monitored by ^18^F-FDG PET imaging will further strengthen our novel finding, the association of FGFRi-regulated HK2 with ^18^F-FDG uptake. ^18^F-FDG PET as a novel biomarker for the response/resistance to FGFR-targeted therapy in cancers is thus indicated.

Since ^18^F-FDG PET/CT imaging can provide both metabolic and anatomical information, it has been widely used in diagnosis, staging, molecular stratification and monitoring of the therapeutic effects and prognostic evaluation of cancer patients 45,46.^ 18^F-FDG PET/CT imaging has been reported to monitor the therapeutic response to tyrosine kinase inhibitors (TKIs), such as EGFR- 47,48, VEGFR- 49, and ALK-TKIs 50. The most successful application example of^ 18^F-FDG PET/CT imaging is prediction the survival outcomes and guidance the targeted therapy in thousands of non-small cell lung cancer with EGFR mutations involving hundreds of research articles 51-53. However, no paper on ^18^F-FDG PET/CT imaging for the drug sensitivity/resistance of FGFR-TKIs is published till now. Further literature search revealed that one related report on ^18^F-FDG PET imaging was used to determine whether Dovitinib (a multitarget-tyrosine kinase inhibitor targeting FGFRs 1-3, VEGFRs, FLT3, c-Kit, PDGFR, and other receptor tyrosine kinases) altered tumor glucose metabolism and subsequent clinical outcome in a phase II study of 15 patients with recurrent or metastatic adenoid cystic carcinoma. ^18^FDG-PET scans detected an early metabolic response only in 3 of 15 patients, but it did not correlate with RECIST (Response Evaluation Criteria in Solid Tumors) response. The authors claimed that they could not determine whether the observed effects were due to the specific inhibition of FGFR or other target receptors, or a combinatorial effect, because the enrolled patients were not selected for FGFR aberrance and Dovitinib was a multitarget kinase inhibitor 54. Selective FGFR inhibitors in the selected patients with right drug target would be required to determine whether ^18^FDG-PET could respond to the FGFR signaling inhibition in this rare cancer type.

## Conclusions

We demonstrated that FGFR-TKI treatment response/resistance in cancer cells visualized by ^18^F-FDG PET imaging was correlated with FGF/FGFR signaling-mediated glucose metabolism via mTOR/HK2. This study had revealed the novel association between the molecular mechanism (FGFR/mTOR/HK2 axis) and radiological phenotype (^18^F-FDG PET) of FGFR-targeted therapy in multiple preclinical models. Considering ^18^F-FDG PET imaging technology is routinely used in clinic practice, the expedient adoption of ^18^F-FDG PET biomarker-based imaging strategy to assess response/resistance to FGFR inhibition would benefit treatment selection and reformulation regimen for cancer patients.

## Supplementary Material

Supplementary figures and table.

## Abbreviations

CTC: circulating tumor cell
^18^F-FDG: ^18^F-fluorodeoxyglucose
FGFR: fibroblast growth factor receptor
FGFRi: fibroblast growth factor receptor inhibitor
GLUT: glucose transporter
HK: hexokinase
HK2: hexokinase 2
IHC: immunohistochemistry
LIHC: liver hepatocellular carcinoma
OS: overall survival
PET: positron emission tomography
RECIST: response evaluation criteria in solid tumors
ROI: region of interest
SNP: single-nucleotide polymorphism
STR: short tandem repeats
SUVmean: mean standardized uptake value
TMT: tandem mass tag
TV: tumor volume
