# Supplementary material for: 18F-FDG PET as an imaging biomarker for the response to FGFR-targeted therapy of cancer cells via FGFR-initiated mTOR/HK2 axis
Source: Theranostics. 2022 Aug 29;12(14):6395–408. doi: 10.7150/thno.74848 (PMC9475468; doi:10.7150/thno.74848)
Supplement: Supplementary file 1 — Supplementary figures and table. [file thnov12p6395s1.pdf]

Supplementary Information

^{18}F-FDG PET as an Imaging Biomarker for the Response to FGFR-Targeted Therapy of Cancer Cells via FGFR-initiated mTOR/HK2 Axis

Yuchen Jiang*, Qinghe Zeng*, Qinghui Jiang, Xia Peng, Jing Gao, Haiyan Wan, Luting Wang, Yinglei Gao, Xiaoyu Zhou, Dongze Lin, Hanyi Feng, Sheng Liang, Hu Zhou, Jian Ding[#], Jing Ai[#], Ruimin Huang[#]

*These authors contributed equally to this article.

#Corresponding authors:

Ruimin Huang, Ph.D., Shanghai Institute of Materia Medica, Chinese Academy of Sciences, Shanghai 201203, China. Phone: 86-21-5081-7066; E-mail: rmhuang@simm.ac.cn

Jing Ai, Ph.D., Shanghai Institute of Materia Medica, Chinese Academy of Sciences, Shanghai 201203, China. Phone: 86-21-5080-6072; E-mail: jai@simm.ac.cn

Jian Ding, M.D., Shanghai Institute of Materia Medica, Chinese Academy of Sciences, Shanghai 201203, China. Phone: 86-21-5080-6079; E-mail: jding@simm.ac.cn

Supplementary methods

Mass spectrum

1) Protein extraction and filter-aided sample preparation

The NCI-H1581 cells were washed three times with cold PBS and the cells were lysed by SDT lysis buffer (2% SDS (m/v), 100 mM DTT, 100 mM Tris, pH=7.6). The lysates were incubated for 10 min at 95℃ and centrifuged at 15,000×g for 30 min. The supernatants were collected, and protein concentration was determined by tryptophan fluorescence emission assay as described previously [1]. Filter aided sample preparation [2,3] was performed with slight modifications, using 50 mM triethylammonium bicarbonate (TEAB) instead of ammonium bicarbonate buffer for compatibility with TMT labeling.

2) TMT labeling

Peptides were reconstituted in 100 mM TEAB and their concentration was determined by BCA assay. 3 NCI-H1581 control samples, 3 NCI-H1581 with AZD4547 treatment samples and 2 mixture samples were labelled by eight of TMT 10-plex reagents (0.8 mg per sample) (Thermo Fisher Scientific, USA) with reporters at m/z = 126, 127N, 127C, 128N, 128C, 129N, 129C, 130N, respectively. Each sample containing 50 μg of peptide in 50 μl TEAB buffer was combined with 41 μl of its respective 10-plex TMT reagent and incubated for 1 h at room temperature. Then, 8 μl of 5% hydroxylamine was added to the sample and incubated for 15 min to quench the reaction. Equal amounts of each TMT-labelled sample were combined in new microcentrifuge tubes and dried using a SpeedVac.

3) High pH reversed-phase liquid chromatography peptides fractionation

Half of the TMT-labeled peptides mixture was fractionated using a Waters XBridge BEH130 C18 3.5 μm 2.1×150 mm column on an Agilent 1290 HPLC operating at 0.2 ml/min. Buffer A was consisted of 10 mM ammonium formate and buffer B was consisted of 10 mM ammonium formate with 90% acetonitrile. Both buffers were adjusted to pH 10 with ammonium hydroxide as described previously [4]. A CBS-B programed multifunction automatic fraction-collecting instrument (Huxi Instrument, China) was coupled to the HPLC and used to collect eluted

peptides. The total number of fractions collected was 28 and concatenated to 14 (pooling equal interval RPLC fractions). Ammonium hydroxide and ammonium formate were evaporated in a SpeedVac.

4) Nanoflow liquid chromatography tandem mass spectrometry

All experiments were performed on an Orbitrap Fusion mass spectrometer with a nanoLC easy1200 (Thermo Fisher Scientific). Peptides were loaded on a self-packed column (75 μm×150 mm, 3 μm ReproSil-Pur C18 beads, 120 Å; Dr. Maisch, Germany) and separated with a 90-min gradient at a flow rate of 300 nl/min. Solvent A was 100% H_2O and 0.08% formic acid; solvent B was 80% acetonitrile and 0.08% formic acid. The Orbitrap Fusion was programmed in the data-dependent acquisition mode. An MS1 survey scan of 375-1,500 m/z in the Orbitrap at a resolution of 120,000 was collected with an AGC target of 400,000 and maximum injection time of 50 ms. Precursor ions were filtered according to monoisotopic precursor selection, charge state (+2 to +7), and dynamic exclusion (45 sec with a ±10 ppm window). Then, the most intense precursors were subjected to HCD fragmentation with a duty cycle of 3 sec. The instrument parameters were set as below: 38% normalized collision energy with 5% stepped collision energy, 50,000 resolution, 100,000 AGC target, 105 ms maximum injection time, 105 Da first mass, and 1 m/z isolation width.

5) Data analysis

Raw files were processed by search against the UniProt/SwissProt Human database containing 75074 sequence entries using Maxquant (1.6.7.0) [5], with default settings for 10-plex TMT quantification. Trypsin/P was selected as the digestive enzyme with allowance of one missed cleavage. Minimum 7 amino acids for peptide, > 2 peptides were required per protein. For peptide and protein identification, false discovery rate (FDR) was set to 1%. TMT reporter ion intensity was used for quantification.

Supplementary References

1. Wiśniewski JR, Zougman A, Nagaraj N, Mann M. Universal sample preparation method for proteome analysis. Nat Methods. 2009; 6: 359-62.
2. Chen X, Yu C, Gao J, Zhu H, Cui B, Zhang T, et al. A novel USP9X substrate TTK contributes to tumorigenesis in non-small-cell lung cancer. Theranostics. 2018; 8: 2348-60.
3. Wang Y, Yang F, Gritsenko MA, Wang Y, Clauss T, Liu T, et al. Reversed-phase chromatography with multiple fraction concatenation strategy for proteome profiling of human MCF10A cells. Proteomics. 2011; 11: 2019-26.
4. Cox J, Mann M. MaxQuant enables high peptide identification rates, individualized p.p.b.-range mass accuracies and proteome-wide protein quantification. Nat Biotechnol. 2008; 26: 1367-72.
5. Kulak NA, Pichler G, Paron I, Nagaraj N, Mann M. Minimal, encapsulated proteomic-sample processing applied to copy-number estimation in eukaryotic cells. Nat Methods. 2014; 11: 319-24.

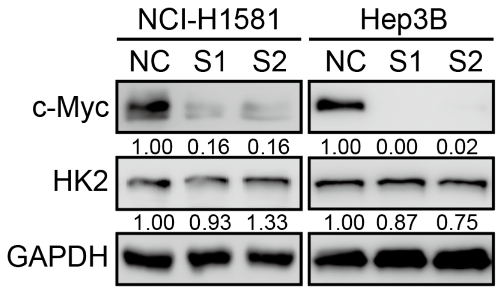

Supplementary Figure S1. Western blot analysis on protein levels of HK2 in NCI-H1581 and Hep3B cells transiently transfected with two siRNAs targeting c-Myc gene (S1 and S2) or siRNA control (NC) for 48 h. GAPDH was used as the loading control. Relative band intensity of target protein was normalized to its corresponding loading control as fold of the control-treated group.

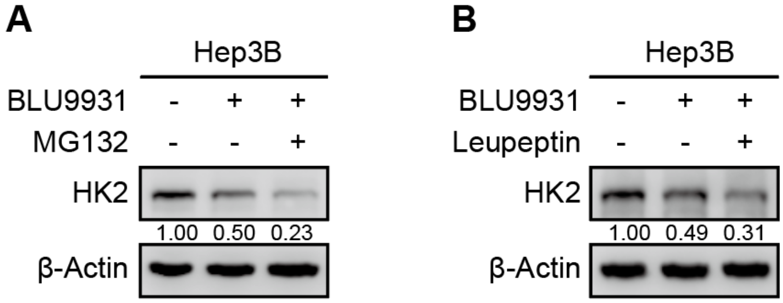

Supplementary Figure S2. Western blot analysis on protein levels of HK2 in Hep3B cells with BLU9931 treatment (0.5 μM) for 24 h. **A**, MG132 (10 μM) or **B**, Leupeptin (10 μM) was added 6 h before sample collection. β-Actin was used as the loading control. Relative band intensity of target protein was normalized to its corresponding loading control as fold of the vehicle-treated group.

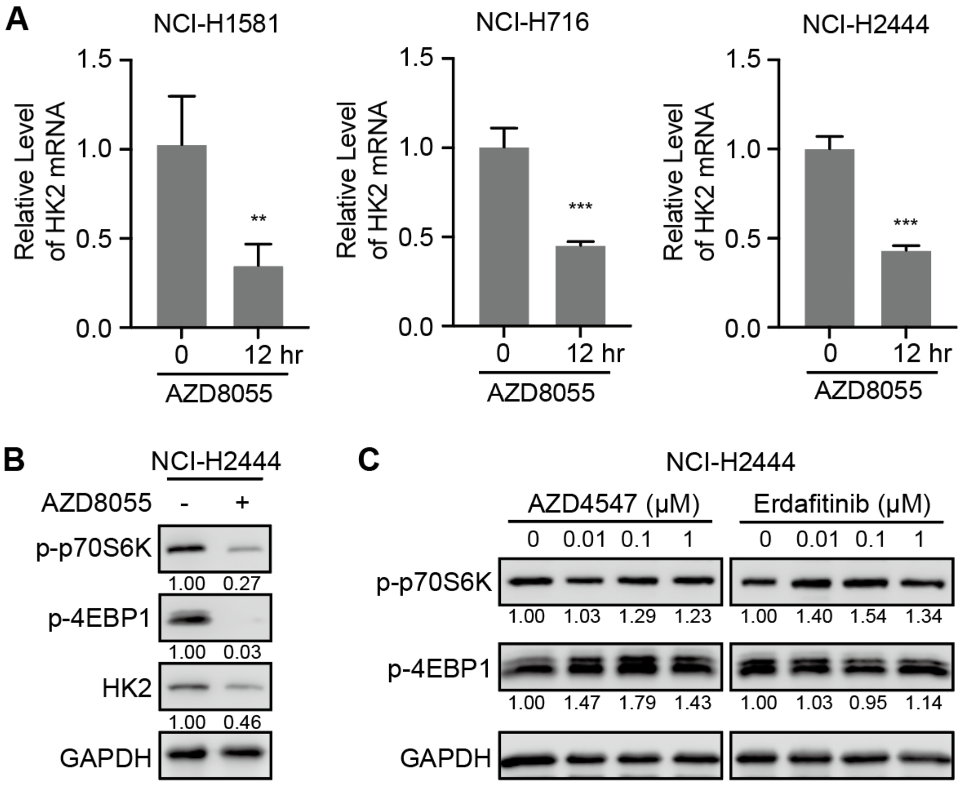

Supplementary Figure S3. A, Quantitative RT-PCR analysis for mRNA levels of *HK2* gene in NCI-H1581, NCI-H716 and NCI-H2444 cells with mTOR inhibitor AZD8055 (0.1 μM) at the indicated time points. Data were shown as mean ± SD; **, $p < 0.01$; ***, $p < 0.001$ vs cells without treatment (0 h) as the normalization controls, using Student's t-test. **B,** Western blot analysis on protein levels of HK2 and mTOR signaling (p-p70S6K and p-4EBP1) with mTOR inhibitor AZD8055 (0.1 μM) for 24 h in NCI-H2444 cells. **C,** Western blot analysis on protein levels of mTOR signaling (p-p70S6K and p-4EBP1) with FGFR inhibitors treatment as indicated for 24 h in NCI-H2444 cells. GAPDH was used as the loading control. Relative band intensity of target protein was normalized to its corresponding loading control as fold of the vehicle-treated group.

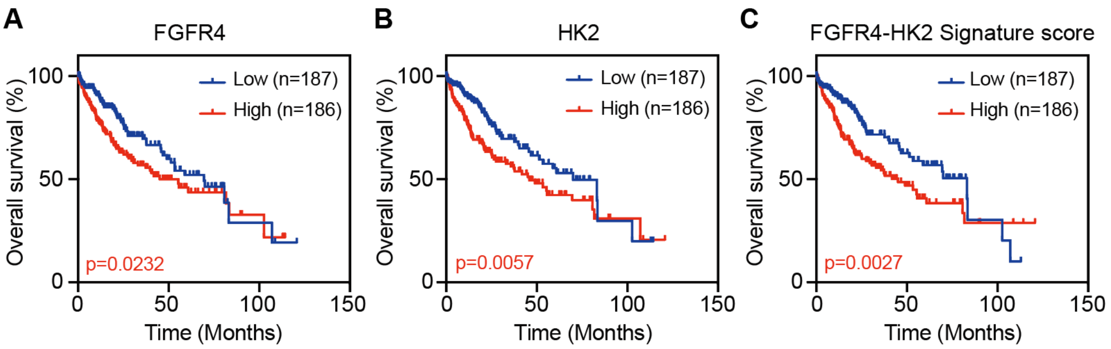

Supplementary Figure S4. Kaplan-Meier plots of overall survival based on the levels of FGFR4 mRNA (**A**), HK2 mRNA (**B**), and FGFR4-HK2 signature score (**C**) in human liver hepatocellular carcinoma patients from TCGA public database (TCGA-LIHC, n = 373). Log-rank test was used for differences in the survival rates between curves.

Supplementary Table S1. List of the differentially expressed proteins upon AZD4547 treatment in NCI-H1581 cells (related to Figure 1)

Uniprot	Gene	Vehicle_1	Vehicle_2	Vehicle_3	AZD4547_1	AZD4547_2	AZD4547_3	Fold change (AZD/Veh)	p value
K7EPJ1	UBE2S	91027.0	73269.9	92222.5	40335.2	41284.6	44329.3	0.5	0.005
Q99622	C12orf57	443590.0	509361.3	467339.4	250839.1	258232.6	251614.2	0.5	0.003
P31350	RRM2	5971900.0	5021814.0	5016820.4	3241955.9	2830329.8	2968515.9	0.6	0.002
P04818	TYMS	1063400.0	926186.3	1041550.1	599661.0	546231.3	573736.6	0.6	0.001
Q8NEN9	PDZD8	3627.6	5113.4	4604.1	2464.7	3041.1	2121.7	0.6	0.018
A0A1C7CYW6	RAB34	384140.0	336056.3	360751.2	218912.2	201782.7	209870.9	0.6	0.001
O00762	UBE2C	2144300.0	2463692.5	2551264.0	1312587.1	1429821.4	1446669.4	0.6	0.002
H3BVE5	SPATA2L	19401.0	23202.0	21522.7	11226.7	13545.3	12806.8	0.6	0.002
A0A499FJK2	TGFB1	148800.0	137282.1	151641.8	87448.5	91894.5	82760.5	0.6	0.000
H0YMA4	KIAA0101	1021900.0	1133457.0	1086986.0	635172.1	630346.8	683351.1	0.6	0.000
Q5R3I4	TTC38	711070.0	699988.6	723747.0	433076.4	430253.9	422711.6	0.6	0.000
Q5SZK8	FREM2	13601000.0	13125429.6	13469390.7	8333529.4	7973976.5	7925118.2	0.6	0.000
O43463	SUV39H1	306670.0	317052.2	332461.8	189260.1	195075.2	192717.8	0.6	0.001
Q9UKT4	FBXO5	221730.0	286599.3	265152.1	136357.2	175571.0	170366.3	0.6	0.013
A0A2R8Y653	UCK2	1863200.0	1985787.0	2076644.9	1221924.7	1252763.5	1223810.1	0.6	0.003
P14635	CCNB1	1101200.0	1156961.4	1183965.6	739780.9	712999.0	712393.4	0.6	0.000
P29317	EPHA2	809350.0	876811.6	863928.0	558416.6	560392.2	500470.4	0.6	0.001
Q9NPD8	UBE2T	1115500.0	1193118.0	1198117.8	735019.7	732163.3	765788.9	0.6	0.000
P20248	CCNA2	774440.0	618366.1	718185.4	495065.3	402852.7	449167.3	0.6	0.007
P41440	SLC19A1	969890.0	970109.1	1093938.0	701161.8	671885.3	565114.7	0.6	0.008
Q7L590	MCM10	330590.0	354319.7	349101.8	235470.8	215268.6	215951.7	0.6	0.000
E9PRJ3	CD151	539930.0	520131.1	559830.1	359005.7	350811.5	342336.1	0.6	0.000
H0YAN3	MSRA	262860.0	308231.6	312087.6	190100.0	197746.9	186682.5	0.7	0.011
Q9UBZ4	APEX2	229160.0	246821.9	244331.0	139557.9	168651.0	163100.7	0.7	0.010
Q9UJQ4	SALL4;SALL3;SALL1	150740.0	165909.1	169185.5	114713.3	99895.4	105056.5	0.7	0.002
A0A0B4J1V9	HELLS	4161700.0	4360245.1	4322470.2	2800282.8	2804981.8	2859002.3	0.7	0.000
E2QRF9	GMNN	456550.0	477653.5	393961.7	293201.1	316632.0	267103.4	0.7	0.006
Q8TBM8	DNAJB14	204660.0	235121.1	244266.5	136978.8	161679.2	153515.8	0.7	0.005
Q5T230	UTF1	88598.0	87583.4	85876.4	58621.7	64351.6	52619.0	0.7	0.018
Q9NYZ3	GTSE1	21239.0	19974.1	23786.1	14271.9	13951.3	15451.4	0.7	0.005
A3KFJ2	AURKA	301760.0	315694.4	376259.0	196501.2	247706.4	228098.0	0.7	0.015
P56178	DLX5	1476700.0	1328949.7	1285960.6	1024067.9	879813.8	870764.7	0.7	0.005

Q9Y6H1	CHCHD2;CHCHD2P9	2759200.0	3114820.8	3182649.4	2002639.3	2073148.9	2079801.5	0.7	0.009
O00311	CDC7	143580.0	138228.5	160947.4	100370.0	106607.6	95728.7	0.7	0.004
P53350	PLK1	282040.0	330640.5	352543.0	219044.5	217709.0	226358.5	0.7	0.027
Q01581	HMGCS1	24521000.0	30903391.9	25034445.3	16471318.0	19921960.5	18962703.3	0.7	0.019
P51530	DNA2	199350.0	220000.1	222487.0	154608.7	144568.2	143587.9	0.7	0.002
Q86U90	YRDC	106890.0	106608.1	114260.2	80610.8	70662.7	75535.2	0.7	0.003
O95229	ZWINT	637300.0	714801.0	721909.7	490456.2	452868.8	501483.8	0.7	0.003
D6RDM5	STARD4	43712.0	39282.7	37139.3	29853.8	29091.1	24907.3	0.7	0.009
O43663	PRC1	480280.0	463581.7	450545.5	329565.2	319832.3	326428.3	0.7	0.001
P08754	GNAI3	764720.0	720510.0	818094.8	557113.8	521402.6	535366.5	0.7	0.003
A0A0C4DFX7	WDR76	507630.0	483933.4	461797.7	347016.6	336004.0	340848.7	0.7	0.003
Q6PI26	SHQ1	33783.0	28366.3	27921.5	25050.2	19506.1	18921.4	0.7	0.036
A0A087WY94	TAF9;AK6	383590.0	356654.7	408108.9	275207.5	266988.7	267562.2	0.7	0.009
Q9Y247	FAM50B	261920.0	252813.7	241450.9	187666.4	171690.9	174319.3	0.7	0.001
Q9C0C4	SEMA4C	77218.0	72267.0	78368.3	57895.0	50285.2	52906.4	0.7	0.004
E7EUW9	ZEB2	1080300.0	1192398.0	1156753.8	814836.9	805941.4	804461.5	0.7	0.006
O60671	RAD1	507440.0	417529.6	464965.8	338657.9	326771.1	318129.1	0.7	0.018
Q9NRL2	BAZ1A	476410.0	506578.8	496219.8	346500.8	368545.6	333184.7	0.7	0.001
Q9NQW6	ANLN	613750.0	720407.1	724740.1	491514.2	485906.1	481920.6	0.7	0.023
A0A494BZT8	TACC3	675310.0	625309.5	661974.0	512979.6	438769.3	442587.4	0.7	0.011
Q9ULX3	NOB1	2251000.0	2124087.4	2253571.8	1557395.3	1538057.5	1634587.1	0.7	0.000
O75419	CDC45	318150.0	337640.4	372932.0	237778.7	250269.5	247182.2	0.7	0.012
Q9Y5N6	ORC6	1326400.0	1582663.5	1557831.2	1015867.9	1105773.8	1072649.0	0.7	0.016
Q5T179	CKS1B;CKS2	1153300.0	1369015.2	1311782.1	851141.5	981630.4	928849.3	0.7	0.009
Q9H8V3	ECT2	220090.0	208772.5	187757.1	157128.2	145734.1	142251.8	0.7	0.007
A0A6I8PS19	GNL3L	701760.0	741031.3	754087.2	516953.9	549063.5	522206.7	0.7	0.000
Q9H6F5	CCDC86	561880.0	580255.1	587389.6	397241.3	439331.0	416035.9	0.7	0.003
Q9ULW0	TPX2	1795600.0	2019217.7	1979019.7	1402522.0	1433361.6	1366702.2	0.7	0.007
O60427	FADS1	1151900.0	1014803.5	1045274.4	815961.1	735278.7	780511.7	0.7	0.003
Q9H5Q4	TFB2M	318170.0	307249.2	299380.5	235966.8	219266.7	218760.1	0.7	0.001
H3BU04	TIPIN	294250.0	282736.8	276200.7	224810.9	198945.8	200079.2	0.7	0.007
O15525	MAFG	188100.0	205393.4	202832.9	148624.1	154268.3	134275.2	0.7	0.005
P04183	TK1	2427800.0	2652807.7	2408550.5	1780711.6	1836898.7	1874892.0	0.7	0.003
Q9NSI2	FAM207A	322480.0	321002.1	312465.0	230418.6	236812.0	235172.0	0.7	0.000

Q9NPF0	CD320	865480.0	859993.4	773602.3	602616.9	615666.7	627081.7	0.7	0.009
Q96HE9	PRR11	324940.0	291387.6	320772.6	235523.7	234031.7	222667.7	0.7	0.004
Q9NRX1	PNO1	846310.0	885040.7	969845.8	688266.7	661736.7	652695.4	0.7	0.010
A0A494C0R8	CLUH	9023600.0	9162599.1	9150744.0	6932263.8	6665513.7	6676702.9	0.7	0.001
Q9P0R6	GSKIP	664160.0	733676.5	608642.7	538492.0	465717.4	485924.0	0.7	0.014
Q5VT82	PCDH9	218710.0	241750.7	235144.5	155600.7	188485.7	173033.6	0.7	0.017
Q6FIF0	ZFAND6	673740.0	638733.2	682780.2	482580.3	491664.8	509097.3	0.7	0.000
Q2T9J0	TYSND1	349720.0	329179.8	335381.6	253788.4	253823.9	247378.8	0.7	0.001
O95149	SNUPN	1598500.0	1500578.1	1562151.4	1202813.6	1114600.8	1153069.9	0.7	0.001
Q99661	KIF2C	1769700.0	1774813.2	1867143.1	1417004.2	1296520.5	1317239.6	0.7	0.002
H7C4H8	SLBP	289440.0	266756.9	261403.0	225201.0	195802.1	189576.6	0.7	0.014
O60488	ACSL4	1163200.0	1212919.3	1231487.1	884338.1	934097.1	879588.3	0.7	0.000
K7EQH5	AES	132050.0	135867.7	157685.0	110884.5	105701.3	101769.0	0.7	0.021
G3V529	DDX24	804910.0	822756.6	849775.8	598973.3	630818.8	625871.6	0.7	0.000
P17844	DDX5	20890000.0	23195802.3	18736481.0	15258519.0	16092865.8	15718638.5	0.7	0.037
P08243	ASNS	11216000.0	12446528.1	12303450.4	8698559.4	9163960.2	9121600.3	0.8	0.005
Q99808	SLC29A1	1877500.0	2042310.7	2027087.4	1482934.4	1513700.8	1469257.8	0.8	0.005
Q9H9Y2	RPF1	401700.0	387411.0	430826.8	315651.8	306318.1	295545.7	0.8	0.003
Q9NZM5	GLTSCR2	380060.0	336796.9	364892.6	303173.3	257609.5	256051.2	0.8	0.018
Q9NYP9	MIS18A	648930.0	791331.7	763522.0	500825.1	586400.9	577316.5	0.8	0.026
H0Y997	TXNDC15	69683.0	56004.2	63645.0	45362.9	46365.5	51277.8	0.8	0.028
J3KPS2	FAM83H	660130.0	556390.7	665152.0	513872.3	464258.9	443565.6	0.8	0.021
O43709	WBSCR22	608520.0	520079.7	597618.9	479412.7	387884.7	438412.6	0.8	0.025
P04035	HMGCR	610580.0	598513.4	665847.2	499019.8	456796.1	465014.6	0.8	0.003
Q71RC2	LARP4	2326500.0	2296075.8	2287835.0	1786134.2	1705910.8	1750201.7	0.8	0.001
X6RF82	CDC123	2711800.0	2578642.9	2313805.4	2140914.3	1983227.5	1646688.1	0.8	0.048
E9PKK4	NCAPD3	134900.0	109966.6	111737.6	94967.3	85054.8	91095.0	0.8	0.036
A0A3B3IRT5	ITPR1;ITPR2	15619.0	14467.8	18579.1	12038.7	13708.7	11323.0	0.8	0.048
P26358	DNMT1	8832100.0	8921383.3	8799174.3	6715361.9	6724045.2	6798216.6	0.8	0.000
F5H2P0	TSPAN11	445830.0	494703.2	456563.9	309058.7	368932.7	389045.8	0.8	0.042
Q9Y4D7	PLXND1	119400.0	106057.8	95882.2	87620.4	81396.6	76407.5	0.8	0.031
O75362	ZNF217	299070.0	324422.4	298000.0	237706.0	242249.7	226030.7	0.8	0.002
Q14680	MELK	446610.0	374002.7	423194.6	336250.8	303226.4	313460.2	0.8	0.019
P12036	NEFH	242880.0	210135.5	228629.6	192685.6	159852.4	170502.5	0.8	0.021

A0A494C100	MCM8	392320.0	361227.0	368269.3	296758.8	283094.3	281049.8	0.8	0.002
Q9NR30	DDX21	21029000.0	22578619.1	21500375.2	16671026.5	16845751.1	16453771.7	0.8	0.003
E5RK48	RDH10	821930.0	783051.2	774396.9	623110.2	607453.4	597787.3	0.8	0.001
B8ZZA8	GLS	426450.0	476948.9	507789.8	342890.1	381833.2	360427.0	0.8	0.018
C9J7E8	MKLN1	553340.0	520439.7	582175.6	421867.6	423371.8	429463.0	0.8	0.013
P60606	CTXN1	196930.0	186044.7	221776.9	161261.3	149548.1	154993.1	0.8	0.024
O75534	CSDE1	25154000.0	25961297.4	26244580.1	19706991.7	19875701.7	20040192.7	0.8	0.001
Q9NTW7	ZFP64	706820.0	626183.8	679304.2	573527.0	463428.1	514845.3	0.8	0.031
Q8WVX9	FAR1	610560.0	625052.3	601492.1	483618.5	464381.6	468881.8	0.8	0.000
Q9BVP2	GNL3	1706400.0	1926125.9	1940088.8	1388436.6	1471171.1	1441778.6	0.8	0.015
J3KMZ9	LDLR	673850.0	692633.9	744702.1	547267.3	549393.9	534458.9	0.8	0.009
P60228	EIF3E	11210000.0	10632523.7	10926965.6	8574898.9	8333662.2	8446972.0	0.8	0.001
Q9GZU7	CTDSP1	113180.0	98379.0	97183.2	91052.5	76114.6	72489.8	0.8	0.045
Q15398	DLGAP5	2107800.0	2027704.0	2122577.3	1661547.8	1620143.2	1586838.8	0.8	0.000
Q5T3I0	GPATCH4	933610.0	924231.9	948245.1	757900.2	736836.4	695653.7	0.8	0.006
A0A087WT20	DCAF13	2320000.0	2154432.3	2239767.2	1825811.3	1709828.6	1711024.9	0.8	0.001
Q15014	MORF4L2	835450.0	798480.8	847193.6	673520.1	618357.3	648712.1	0.8	0.002
O95758	PTBP3	38530.0	38061.2	44724.8	33486.2	32799.8	28602.6	0.8	0.027
Q9H467	CUEDC2	722210.0	820596.5	828721.3	632004.5	617602.0	608930.2	0.8	0.026
Q8NFA0	USP32	668150.0	636881.6	662321.6	526383.9	495393.9	520643.7	0.8	0.001
Q6PL18	ATAD2	2176200.0	2247832.7	2124166.4	1713524.9	1716201.0	1710671.9	0.8	0.004
M0R253	DHPS	80334.0	69654.3	70904.9	63185.9	57181.5	53148.4	0.8	0.023
Q9UHE8	STEAP1	813980.0	852381.4	813327.7	668957.2	634217.4	646997.8	0.8	0.000
K7ES31	EIF3K	2939800.0	3393941.9	3448859.2	2490536.3	2615509.6	2589554.3	0.8	0.034
F8VXG3	TGIF1;TGIF2	332010.0	314994.9	334522.6	236608.2	262660.2	273562.3	0.8	0.018
Q96BY7	ATG2B	1013900.0	1059755.0	1025808.9	807893.4	837189.6	797049.7	0.8	0.000
F8WA97	TUT1	72804.0	74977.5	65139.7	54233.4	54358.8	59203.9	0.8	0.014
J3KRU4	RNF138	160390.0	149867.5	156950.1	114872.0	126305.4	127619.7	0.8	0.007
Q13283	G3BP1	5751300.0	5809751.3	5554602.9	4615778.1	4405111.0	4496665.1	0.8	0.000
Q96FF9	CDCA5	776720.0	649225.3	696584.7	621457.0	521213.8	533954.7	0.8	0.036
B4DZ85	NCOA4	66831.0	75594.7	79624.6	55044.8	57441.1	63121.6	0.8	0.026
Q9Y5J1	UTP18	2993900.0	3218096.1	3099325.4	2446230.1	2458937.8	2467536.3	0.8	0.007
F8W9J4	DST	24996000.0	25937124.4	25115385.8	20258504.4	20205177.6	19829434.4	0.8	0.000
Q9Y620	RAD54B	196650.0	214944.3	205921.5	168641.2	168632.2	152593.1	0.8	0.006

H0YMP3	CCNB2;CCNB2V	510790.0	463082.8	492292.0	394814.3	376895.8	391486.1	0.8	0.006
H7BYV1	IFITM2;IFITM3;IFITM1	1012700.0	1219554.0	1217483.9	844462.5	936646.0	959555.4	0.8	0.045
Q6ZTW0	TPGS1	169070.0	152444.3	160654.5	139538.0	120249.2	123379.3	0.8	0.019
D6RG30	ASTE1	26698.0	29021.0	24326.8	22127.3	22467.6	19029.8	0.8	0.035
P18827	SDC1	565990.0	505647.9	487073.0	420743.4	428922.8	389282.7	0.8	0.018
P08238	HSP90AB1	111270000.0	115819572.4	106975514.4	79704832.3	88240997.3	97659762.5	0.8	0.044
G3V5T9	CDK2	244830.0	284182.0	250483.5	199695.2	219431.9	201349.8	0.8	0.020
E9PB90	HK2	612420.0	568220.0	563951.6	483029.9	433265.5	473863.4	0.8	0.007
P52732	KIF11	8182300.0	8839092.2	8326442.1	6714700.6	6876982.4	6627290.6	0.8	0.004
Q02241	KIF23	1186600.0	1188180.5	1215398.4	963163.4	934144.3	967068.1	0.8	0.000
Q9NS91	RAD18	1188600.0	1354614.3	1217632.9	1026316.3	996829.7	979875.0	0.8	0.022
O15294	OGT	3091100.0	2888314.6	3171625.6	2510837.8	2329696.4	2467183.4	0.8	0.004
Q15058	KIF14	1315400.0	1243264.1	1312973.9	1088344.9	999520.2	1004278.6	0.8	0.004
Q7Z417	NUFIP2	1159500.0	1195535.3	1206410.5	1001848.7	923098.8	920076.1	0.8	0.007
Q14152	EIF3A	52868000.0	51355300.5	52184267.8	42287933.4	40525530.0	42138556.4	0.8	0.000
Q8NEM2	SHCBP1	183220.0	209040.0	203796.2	147182.5	168438.6	160650.3	0.8	0.017
Q7Z4L5	TTC21B	694540.0	684147.6	703586.3	572263.9	541275.0	552307.8	0.8	0.001
Q6PIW4	FIGNL1	296160.0	300923.1	283986.9	223554.4	235325.1	246199.0	0.8	0.005
B0QY89	EIF3L	12021000.0	12337492.4	12215558.0	9741407.8	9934782.7	9615722.7	0.8	0.000
P14373	TRIM27	117460.0	108830.0	112988.9	99199.6	85238.9	87394.1	0.8	0.026
O95478	NSA2	1161100.0	1206387.4	1221009.6	949408.7	966431.0	965152.1	0.8	0.002
P11169	SLC2A3;SLC2A14	1359100.0	1326738.2	1464178.6	1060835.4	1145282.6	1125792.3	0.8	0.005
Q13823	GNL2	1645000.0	1438654.1	1463284.8	1340096.6	1157460.9	1154128.7	0.8	0.029
G3V195	EML3	155170.0	155031.3	145097.0	122199.1	124237.9	119713.8	0.8	0.004
O14745	SLC9A3R1	7196400.0	6299897.6	6638113.0	5810920.7	5187592.6	5204924.0	0.8	0.016
Q14444	CAPRIN1	8709700.0	10136205.6	9549487.6	7168343.0	7907892.5	7777889.9	0.8	0.020
Q3B7T1	EDRF1	112630.0	110110.6	112040.5	97579.4	82293.4	89789.1	0.8	0.045
P04181	OAT	6634800.0	6488652.8	6908742.2	5343986.5	5351386.5	5447951.5	0.8	0.004
A0A087WVR3	UHRF1	3385100.0	3511001.0	3675393.2	2884398.4	2740549.9	2894397.6	0.8	0.003
A0A0A0MTR2	OGFOD1	1112200.0	1137880.1	1019999.1	905102.5	865653.0	865218.4	0.8	0.012
Q13111	CHAF1A	977300.0	1148063.6	1121000.9	823830.4	903084.8	890176.6	0.8	0.031
J3QS74	SKA2	247210.0	218981.7	222343.0	186555.5	178417.3	190393.4	0.8	0.016
H3BRB3	KIF22	351170.0	338190.7	346842.4	281879.9	285171.3	268842.9	0.8	0.001
O14757	CHEK1	472160.0	488047.9	485091.7	384657.0	393643.4	387835.7	0.8	0.000

P11388	TOP2A	37126000.0	42511065.1	39993041.6	30352377.8	33136868.8	33052250.6	0.8	0.014
P11940	PABPC1;PABPC3	35519000.0	36353634.0	35144557.6	28627082.0	28719154.6	29042296.0	0.8	0.001
Q53HL2	CDCA8	388960.0	429004.0	417121.6	312054.4	359562.9	325722.5	0.8	0.017
O94925	GLS	1967000.0	1647262.0	1907216.1	1627756.1	1435391.3	1396803.3	0.8	0.043
Q9NWQ9	C14orf119	122410.0	102462.7	113510.3	99133.4	83766.2	90600.9	0.8	0.040
O60566	BUB1B	884270.0	828311.3	863630.0	724042.3	688547.9	672006.5	0.8	0.002
H0YIM9	CHURC1-FNTB;CHURC1	466210.0	541166.8	478025.6	370776.6	411273.7	420109.9	0.8	0.026
E7EUU4	EIF4G1	42495000.0	41784846.2	40740872.1	34178580.1	33389404.0	33631583.9	0.8	0.000
B1AHQ6	CENPM	364070.0	395393.3	380420.3	308212.3	306280.4	308892.0	0.8	0.012
Q13129	RLF	461070.0	507170.3	473849.4	383843.6	379671.4	405124.9	0.8	0.005
Q9NVI1	FANCI	3231000.0	3558215.5	3281962.9	2754455.6	2715768.5	2693773.8	0.8	0.016
Q9UBI6	GNG12	3135500.0	2841048.6	3050016.3	2565856.8	2377607.3	2382426.3	0.8	0.006
F5H1D6	POLE	2047900.0	2269691.3	2192196.1	1722121.7	1815610.2	1751512.6	0.8	0.009
O14929	HAT1	3635200.0	3910061.4	4043300.0	3060234.4	3126291.6	3230249.5	0.8	0.010
P13995	MTHFD2	1310100.0	1360786.1	1361389.2	1103223.9	1105773.8	1071035.5	0.8	0.000
Q68D10	SPTY2D1	495800.0	415076.3	477012.6	392850.3	366506.5	370415.3	0.8	0.049
P62854	RPS26;RPS26P11	4847000.0	4692546.8	5031717.4	4022472.0	3889939.2	3953886.8	0.8	0.003
A0A2R8YCV2	ZMYM6NB	292510.0	295682.2	276573.1	255706.2	232214.4	216501.2	0.8	0.035
Q9Y320	TMX2	2324900.0	2370806.4	2352984.6	1892998.0	1942208.2	1907413.3	0.8	0.000
A0A499FIY5	FBXW8	403820.0	430773.3	439392.6	339226.6	338406.6	360568.2	0.8	0.004
P98170	XIAP	1608700.0	1599070.3	1525802.6	1309214.5	1285522.3	1265255.9	0.8	0.001
B7ZKQ9	SCARB1	350900.0	392744.5	412702.1	294490.6	322447.4	326498.9	0.8	0.031
Q9Y2Y1	POLR3K	84415.0	93030.1	91914.6	75115.5	73896.1	70891.4	0.8	0.009
B7ZAA0	PMS1	418830.0	453084.5	483085.6	382818.6	359832.0	364359.8	0.8	0.025
P49427	CDC34	845750.0	851918.6	832346.3	693755.4	661783.9	712393.4	0.8	0.007
A0A2U3TZJ9	DDX3X;DDX3Y	28271000.0	31635782.7	31885585.7	23450533.2	25569780.8	26021594.7	0.8	0.017
Q9H7Z6	KAT8	284970.0	261989.1	286166.8	243161.6	218407.6	220903.0	0.8	0.012
B9ZVN9	POLR1A	3834300.0	3662622.3	3762143.6	3194475.5	3030233.8	3001188.5	0.8	0.002
F5H4V9	PDCD2	69280.0	63117.3	70184.8	58380.3	52390.4	55614.0	0.8	0.013
Q6PD74	AAGAB	2795300.0	2725172.5	2681066.7	2155396.4	2251009.2	2346476.3	0.8	0.006
A0A0A6YYI3	PPAN-P2RY11;PPAN	6703200.0	5822609.3	6081957.5	5472143.8	4947330.1	4909912.8	0.8	0.025
F5GYQ2	TRMT112	1124700.0	994282.1	1113105.5	887644.5	899497.4	876361.4	0.8	0.036
Q96SB3	PPP1R9B	2475500.0	2344010.4	2417339.7	2060898.6	1955236.2	1950220.4	0.8	0.001
P52292	KPNA2	5622100.0	5116243.1	5040159.1	4429030.8	4234897.6	4345151.0	0.8	0.020

Q6IN84	MRM1	482030.0	443801.0	470775.7	391798.9	382782.0	377590.2	0.8	0.008
Q9BZE4	GTPBP4	5711000.0	5784549.6	5697614.3	5014467.5	4549457.3	4622565.5	0.8	0.021
H0Y8R1	GRSF1	2621600.0	2669728.8	2823382.9	2063411.5	2201257.4	2431334.3	0.8	0.038
Q15742	NAB2	1017500.0	938478.5	935086.1	868004.3	758596.9	759032.5	0.8	0.029
P55884	EIF3B	26235000.0	24047000.8	25523067.7	21230595.2	20536069.5	20810620.3	0.8	0.010
Q14146	URB2	515080.0	505411.3	505505.6	431687.7	418363.5	409909.8	0.8	0.002
Q8ND90	PNMA1	747380.0	673295.4	718632.3	615445.9	533533.8	619064.8	0.8	0.037
Q5BJF6	ODF2	267690.0	313884.0	310831.3	230636.8	258487.5	248609.1	0.8	0.046
Q2NL82	TSR1	295830.0	289561.8	293724.6	240900.0	239191.0	246904.9	0.8	0.000
S4R3V8	LSR	890850.0	972372.1	974364.5	758098.6	785785.8	803806.0	0.8	0.010
Q86XI2	NCAPG2	1172200.0	1302050.8	1269673.2	990408.4	1059562.2	1047690.7	0.8	0.011
O75131	CPNE3	14328000.0	15948528.4	16003374.1	12282730.9	13115782.4	12913737.6	0.8	0.019
D6RGF1	DKK2	665930.0	730076.3	662222.3	553595.8	576346.7	575299.6	0.8	0.016
Q9NXH9	TRMT1	897120.0	997933.8	1011011.2	762926.0	832233.3	813184.3	0.8	0.019
Q9NXW2	DNAJB12	1628800.0	1563839.4	1663649.8	1420707.4	1297559.0	1307105.0	0.8	0.009
A3KN83	SBNO1	1460100.0	1690361.9	1481657.8	1258361.6	1310823.0	1271508.0	0.8	0.050
Q8IWA0	WDR75	1904900.0	2061031.9	2112348.1	1669086.5	1688162.5	1683898.5	0.8	0.026
P06756	ITGAV	14376000.0	14184927.4	13765841.4	11494477.7	11551480.1	12063645.4	0.8	0.001
Q13895	BYSL	1589900.0	1759743.6	1739277.0	1399149.5	1408580.1	1414400.2	0.8	0.028
Q9UNZ5	C19orf53	2385100.0	2736127.5	2729978.6	2027305.3	2315252.3	2176256.2	0.8	0.037
Q9UKD2	MRTO4	3836100.0	3946012.3	3804947.7	3238054.3	3174532.9	3211291.4	0.8	0.001
J3KPH8	HDAC7	318520.0	309568.8	320057.6	264613.7	264817.4	258204.2	0.8	0.000
Q8TDD1	DDX54	5483000.0	5197711.3	5429467.9	4583904.1	4431355.8	4388613.6	0.8	0.001
P30520	ADSS	1967700.0	2170119.0	2261119.6	1765105.3	1815091.0	1746873.9	0.8	0.040
Q9Y316	MEMO1	161930.0	173670.2	181515.2	136800.3	141939.0	151993.1	0.8	0.016
P49643	PRIM2	3456500.0	3706185.2	3564311.1	2934259.4	3011824.7	2992566.6	0.8	0.006
Q93034	CUL5	2718000.0	3140639.6	2863158.0	3231110.8	3651895.3	3583597.5	1.2	0.033
Q5SSJ5	HP1BP3	14326000.0	15535530.0	15399051.6	17469199.0	17945105.4	18909761.7	1.2	0.006
Q96CV9	OPTN	2676500.0	2922105.3	2782714.0	3113930.8	3481068.2	3464957.7	1.2	0.019
P21980	TGM2	1526900.0	1322315.0	1402505.0	1775223.0	1636664.2	1694386.0	1.2	0.028
P78310	CXADR	2398100.0	2069261.0	2299802.2	2747379.9	2705242.2	2693219.2	1.2	0.048
O15031	PLXNB2	705680.0	723647.3	679502.8	845190.0	849792.8	844798.0	1.2	0.009
H7C2Z6	GCA	1674400.0	1722198.3	1586085.9	1958796.0	2091322.0	1952388.5	1.2	0.005
Q6JQN1	ACAD10	2682500.0	2901635.4	2806996.2	3220397.9	3413992.9	3476604.9	1.2	0.005

Q7Z434	MAVS	4379000.0	4036378.2	3906247.4	4999919.2	4932697.2	4917375.0	1.2	0.029
P22570	FDXR	7196100.0	7090920.8	7230517.9	8527947.5	8719781.4	8758067.4	1.2	0.000
Q9NXG2	THUMPD1	3662900.0	4243700.3	3736421.4	4395966.5	4886438.5	4793441.0	1.2	0.033
Q96EC8	YIPF6	1322000.0	1465604.4	1347187.3	1728403.9	1643508.6	1626872.8	1.2	0.012
O75170	PPP6R2	279190.0	249861.5	237046.4	305917.6	330712.6	290039.8	1.2	0.038
P23634	ATP2B4	1363100.0	1236166.5	1346293.5	1598924.0	1544949.1	1632116.5	1.2	0.012
Q8TDD2	SP7	139320.0	149008.6	150643.7	170955.7	178176.6	182346.3	1.2	0.004
Q96IX5	USMG5	1500300.0	1437728.3	1429369.3	1759352.1	1673246.4	1862538.9	1.2	0.012
H7C0G1	TMEM245	1190700.0	1183757.4	1064441.9	1362844.8	1389982.2	1424181.8	1.2	0.023
Q9UQL6	HDAC5	446260.0	438447.0	454562.8	560215.3	528435.8	538190.0	1.2	0.002
Q5TA58	AGO1	136080.0	141828.7	130453.2	172364.3	149850.1	174011.8	1.2	0.039
Q9UIQ6	LNPEP	6664200.0	6578144.4	6114234.4	7949983.4	8009378.6	7587804.0	1.2	0.006
P30414	NKTR	223310.0	253487.4	237766.4	273296.4	301243.8	295026.4	1.2	0.015
Q9UL54	TAOK2	231210.0	269400.5	251074.4	317754.7	296108.2	302281.9	1.2	0.029
Q96A26	FAM162A	1302700.0	1150223.8	1083261.8	1441802.4	1368882.5	1499308.5	1.2	0.048
P50440	GATM	7478100.0	8688396.6	8345808.2	9285120.3	10462510.5	10145139.6	1.2	0.028
Q9H336	CRISPLD1	228340.0	226218.2	206179.7	282223.7	266195.7	257548.7	1.2	0.010
Q9P1Z2	CALCOCO1	1164500.0	1295467.6	1284619.9	1475395.8	1542211.3	1552603.2	1.2	0.015
O95470	SGPL1	5014500.0	5828266.8	4954948.1	6044288.6	6629639.5	6624265.4	1.2	0.040
B3KT61	RALYL	113110.0	125504.2	129201.9	139538.0	157534.8	152704.0	1.2	0.021
G3V5N8	ZFYVE1	88402.0	86014.8	86000.5	107082.1	100230.5	111323.8	1.2	0.016
Q6PCB7	SLC27A1	61075.0	64526.5	62944.9	73726.8	83964.4	73160.4	1.2	0.034
Q9H7C9	AAMDC	2155500.0	2305385.0	2396881.2	2602955.0	2899670.7	2901153.9	1.2	0.014
O43861	ATP9B	158330.0	143886.0	137787.5	183540.0	178809.1	177793.3	1.2	0.032
P00387	CYB5R3	3816000.0	4389252.7	3654736.0	4432138.9	5045512.0	5084922.8	1.2	0.046
Q53EL6	PDCD4	1300900.0	1276180.6	1216391.5	1542516.3	1536027.8	1582351.3	1.2	0.003
O94991	SLITRK5	290270.0	297281.7	297612.7	358410.5	346893.7	382849.1	1.2	0.014
Q9BWW4	SSBP3	170540.0	165533.7	161046.7	204687.9	206493.6	200018.7	1.2	0.001
A0A5F9ZH58	ANK2	768390.0	828722.8	737998.5	889760.7	1048658.4	933235.9	1.2	0.031
P23743	DGKA	169670.0	180083.8	194917.6	221365.6	223807.6	225103.0	1.2	0.033
Q5T9B7	AK1	2874800.0	3137450.8	3054088.1	3554678.1	3764096.4	3849969.8	1.2	0.004
Q00266	MAT1A	354470.0	311806.1	294270.8	372727.4	433510.9	378437.3	1.2	0.046
Q9UH62	ARMCX3	1036300.0	1125022.1	1041252.2	1273042.2	1356279.3	1325911.9	1.2	0.004
Q9NZ08	ERAP1	3531500.0	3924616.6	3265923.8	4149372.9	4622291.3	4484866.7	1.2	0.036

Q07617	SPAG1	45399.0	37916.7	44582.3	56329.0	52003.4	49904.3	1.2	0.043
Q9HAT2	SIAE	1578300.0	1598195.9	1589313.6	1869654.6	2058657.7	1968371.9	1.2	0.016
Q8TB96	ITFG1	636690.0	716806.9	638287.7	799362.8	814060.3	860025.1	1.2	0.015
P17612	PRKACA	816390.0	899390.2	841731.4	990672.9	1104168.9	1081523.0	1.2	0.008
O95197	RTN3	713740.0	792617.5	664655.5	871905.9	991684.6	835470.2	1.2	0.040
P06732	CKM	63520.0	62891.0	65978.9	87131.1	75260.2	77446.1	1.2	0.030
E9PH60	WNT16	134070.0	130539.4	136888.7	155779.2	171544.6	173507.6	1.2	0.014
Q96JC1	VPS39	256820.0	233639.8	227601.7	319996.4	295201.9	281917.0	1.2	0.013
Q8N9F7	GDPD1	225610.0	260970.8	246406.7	291905.0	308187.4	315945.9	1.2	0.017
O43524	FOXO3	308640.0	379248.8	357702.3	393637.3	466656.8	450337.1	1.3	0.049
P54803	GALC	861880.0	916671.4	878030.5	1061496.7	1204050.2	1067153.1	1.3	0.019
P51688	SGSH	509210.0	588998.5	521097.8	755651.8	639645.7	636913.7	1.3	0.037
Q99541	PLIN2	849810.0	884629.3	871624.8	1052569.3	1125599.0	1092666.0	1.3	0.001
Q02952	AKAP12	36924000.0	39216849.7	36800609.8	44935723.6	48921028.0	48119958.5	1.3	0.003
Q07954	LRP1	5915700.0	6478366.4	6224472.3	7605453.2	7880986.9	7929151.9	1.3	0.005
C9JRY4	SEC22A	156230.0	190735.3	180983.9	214931.3	230005.3	220746.7	1.3	0.048
Q96CM8	ACSF2	1686000.0	1711809.1	1489602.9	2059509.9	2137203.2	1966304.6	1.3	0.017
Q9NSY0	NRBP2	125620.0	122973.8	109572.6	153682.9	161216.6	137355.9	1.3	0.022
A0A494C1M4	ALDH1L2	644330.0	721127.1	638585.7	827467.5	866691.4	837234.9	1.3	0.018
Q03188	CENPC	461900.0	515399.4	500490.3	642142.0	607311.8	617905.1	1.3	0.008
H3BU63	PPCDC	146100.0	152598.5	135344.4	170063.0	173782.0	205933.1	1.3	0.040
F5H3B1	BAD	47919.0	53540.6	45783.0	58058.9	63435.9	65536.8	1.3	0.017
J3KQI6	STRA6	191180.0	178648.8	196476.8	244695.8	227975.6	248175.5	1.3	0.003
Q8N0X4	CLYBL	614160.0	612245.7	574131.2	764182.4	757275.2	774360.4	1.3	0.005
Q9Y5Y5	PEX16	218660.0	243674.2	205320.7	262292.6	290774.2	300779.4	1.3	0.020
U3KQ24	PGPEP1	36414.0	39326.4	37895.0	42602.0	51262.3	51908.1	1.3	0.049
Q00978	IRF9	930000.0	996750.9	913733.7	1180726.6	1251961.0	1219020.1	1.3	0.003
B7Z2U2	TOM1L2	183470.0	148895.4	168892.5	226543.4	209915.8	210808.7	1.3	0.036
P08473	MME	84388.0	76787.9	79579.9	95754.2	103964.3	111792.7	1.3	0.013
P09417	QDPR	2145400.0	1932966.4	1862574.7	2537090.8	2420703.5	2746261.7	1.3	0.010
Q9Y624	F11R	509250.0	605045.3	604670.1	712139.2	755953.6	766898.1	1.3	0.030
Q99985	SEMA3C	42168.0	35968.9	33654.9	55813.2	45138.2	45835.4	1.3	0.047
Q5T7W0	ZNF618	25174.0	27066.1	25418.8	35661.2	31555.6	34786.7	1.3	0.006
O95833	CLIC3	54188.0	57418.6	56156.8	76213.2	67703.0	76972.2	1.3	0.012

Q8WWI5	SLC44A1	48033.0	39210.2	46208.6	62683.3	57153.2	56077.9	1.3	0.027
P27701	CD82	145300.0	146977.0	137633.6	190622.4	186522.0	190171.6	1.3	0.003
G5E9S9	HMGCLL1	167770.0	159670.4	163340.9	221365.6	211327.1	215830.6	1.3	0.000
Q9UFN0	NIPSNAP3A	19290.0	16205.7	17410.6	22336.9	25894.5	22606.1	1.3	0.014
G5E9M0	PHYHD1	67544.0	57650.1	61425.4	84254.5	77516.5	88306.7	1.3	0.009
P42773	CDKN2C	395930.0	342696.1	358387.6	519294.9	475663.1	483594.6	1.3	0.007
A0A087WZK6	FAM69A	68091.0	77091.3	75801.0	93208.3	105753.3	99570.7	1.4	0.005
A0A1W2PQW3	PAX6	146770.0	165739.4	162690.4	197744.4	222811.6	223792.1	1.4	0.005
Q7L5N7	LPCAT2	1646100.0	1608173.7	1472620.3	2193751.0	2116906.0	2119936.3	1.4	0.007
P62256	UBE2H	708780.0	817767.8	664605.8	934728.1	1098174.2	953303.3	1.4	0.019
Q92522	H1FX	12927000.0	14528492.7	12546271.9	17460602.3	18682885.8	18453454.7	1.4	0.009
Q96JG8	MAGED4	354030.0	356541.6	369749.0	461260.4	510168.3	511567.9	1.4	0.005
P05386	RPLP1	20246.0	17485.8	17204.1	26443.5	25541.5	23468.8	1.4	0.010
P08151	GLI1	44927.0	41720.0	42354.2	60450.2	53310.9	63888.0	1.4	0.016
Q53HC0	CCDC92	38269.0	47178.0	47217.6	59921.8	63100.8	60227.5	1.4	0.037
A0A5F9ZHY1	OTX2;OTX1	551630.0	501641.4	441130.6	706716.6	694117.8	667872.0	1.4	0.030
Q9H3Q1	CDC42EP4	66512.0	90047.0	72111.5	97513.3	115444.0	105298.5	1.4	0.045
A0A5S6RJB7	FGFR2	874510.0	737431.1	738197.1	1098727.1	1005231.8	1184330.7	1.4	0.011
Q96QV1	HHIP	110650.0	107425.9	104318.9	152109.1	158639.3	141349.3	1.4	0.003
Q76KP1	B4GALNT4	30590.0	28143.0	24176.4	36380.0	40213.5	39956.9	1.4	0.023
P60763	RAC3	18673.0	19843.5	19956.1	28385.7	23261.6	30778.8	1.4	0.048
P31943	HNRNPH1	9698800.0	10648467.6	8302110.3	12746953.9	14550276.8	13228866.7	1.4	0.023
C9J406	IMMT	11319.0	11055.3	11913.1	15149.4	18406.3	15032.9	1.4	0.026
Q9Y3P4	RHBDD3	52275.0	48469.5	39775.0	63353.9	70176.5	67528.4	1.4	0.034
A0A0A0MT32	LIPA	364900.0	352869.4	270708.7	476443.5	520128.1	426029.3	1.4	0.038
K7ENS5	FAM117A	148110.0	156877.7	160490.6	218541.9	223647.1	227815.6	1.4	0.001
Q13740	ALCAM	389180.0	384808.6	316258.8	527197.2	527114.2	521853.8	1.4	0.031
B7ZBJ4	CAB39L	35541.0	35871.7	40922.6	52586.8	52248.8	59093.0	1.5	0.004
Q53FP2	TMEM35	217460.0	202467.0	173729.1	291468.5	292874.8	290806.2	1.5	0.027
K7ENI6	TMEM256	50167.0	54157.8	63029.3	82059.0	80513.9	87328.6	1.5	0.017
Q9HCU8	POLD4	33689.0	41326.6	38753.1	50714.7	60457.4	60267.8	1.5	0.008
Q96AQ6	PBXIP1	1103300.0	1032959.0	1062108.0	1617109.4	1618821.5	1610839.0	1.5	0.002
P09914	IFIT1;IFIT1B	271050.0	294103.2	241505.6	378599.6	425519.5	420281.4	1.5	0.006
Q86WU2	LDHD	242010.0	261809.1	231296.1	350957.8	402673.3	373294.3	1.5	0.001

A0A087WTF6	NCAM1	3545500.0	3807608.9	3553337.0	5080794.6	5779988.3	5888628.0	1.5	0.004
P19419	ELK1	4516.8	5056.6	4084.3	8237.0	6673.1	6621.7	1.6	0.009
G5EA09	SDCBP	9524.9	12219.2	10040.6	14619.7	20368.5	18557.8	1.7	0.016
F8WEE4	ZFAND2B	4232.3	2841.3	4553.1	7417.6	6260.5	6294.0	1.7	0.048
P53384	NUBP1	5787.2	10492.6	7010.0	16298.1	12755.2	15452.9	1.9	0.045
Q86YB8	ERO1LB	2746.4	2547.9	1442.0	7192.1	5970.7	5418.2	2.8	0.023
